# Unmasking the Iron–Oxo Bond of the [(Ligand)Fe-OIAr]^2+/+^ Complexes

**DOI:** 10.1021/jasms.2c00094

**Published:** 2022-08-03

**Authors:** Guilherme L. Tripodi, Jana Roithová

**Affiliations:** Department of spectroscopy and Catalysis, Institute for Molecules and Materials, Radboud University, Heyendaalseweg 135, 6525 AJ Nijmegen, The Netherlands

## Abstract

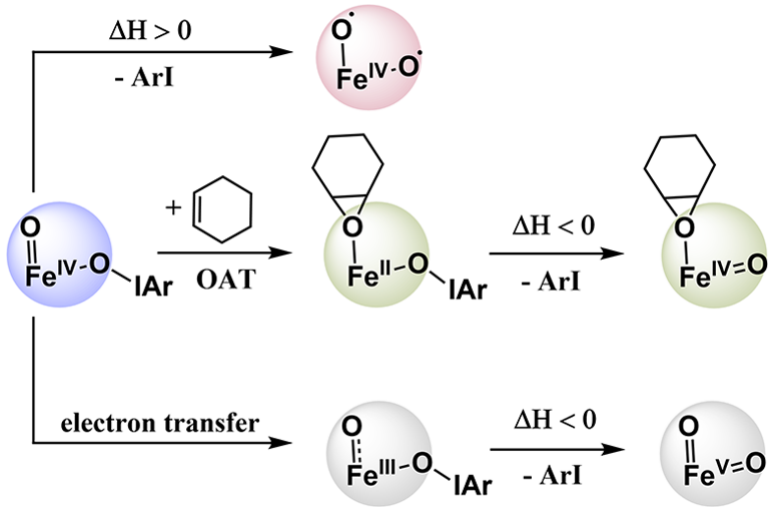

ArIO (ArI = 2-(^t^BuSO_2_)C_6_H_4_I) is an oxidant used to oxidize Fe^II^ species to their Fe^IV^-oxo state, enabling hydrogen-atom transfer (HAT) and oxygen-atom transfer (OAT) reactions at low energy barriers. ArIO, as a ligand, generates masked Fe^n^=O species of the type Fe^(n-2)^-OIAr. Herein, we used gas-phase ion–molecule reactions and DFT calculations to explore the properties of masked iron–oxo species and to understand their unmasking mechanisms. The theory shows that the I–O bond cleavage in [(TPA)Fe^IV^O(ArIO)]^2+^ (**1**^**2+**^, TPA = tris(2-pyridylmethyl)amine)) is highly endothermic; therefore, it can be achieved only in collision-induced dissociation of **1**^**2+**^ leading to the unmasked iron(VI) dioxo complex. The reduction of **1**^**2+**^ by HAT leads to [(TPA)Fe^III^OH(ArIO)]^2+^ with a reduced energy demand for the I–O bond cleavage but is, however, still endothermic. The exothermic unmasking of the Fe=O bond is predicted after one-electron reduction of **1**^**2+**^ or after OAT reactivity. The latter leads to the 4e^–^ oxidation of unsaturated hydrocarbons: The initial OAT from [(TPA)Fe^IV^O(ArIO)]^2+^ leads to the epoxidation of an alkene and triggers the unmasking of the second Fe=O bond still within one collisional complex. The second oxidation step starts with HAT from a C–H bond and follows with the rebound of the C-radical and the OH group. The process starting with the one-electron reduction could be studied with [(TQA)Fe^IV^O(ArIO)]^2+^ (**2**^**2+**^, TQA = tris(2-quinolylmethyl)amine)) because it has a sufficient electron affinity for electron transfer with alkenes. Accordingly, the reaction of **2**^**2+**^ with 2-carene leads to [(TQA)Fe^III^O(ArIO)]^2+^ that exothermically eliminates ArI and unmasks the reactive Fe^V^–dioxo species.

## Introduction

Nonheme iron–oxo species are in the active core of many enzymes involved in the activation of strong C–H bonds and other oxygenation reactions.^1−5^ Understanding their reactivity is crucial for the development of catalysts able to efficiently perform such oxidative transformations.^6−9^

**Figure 1:**
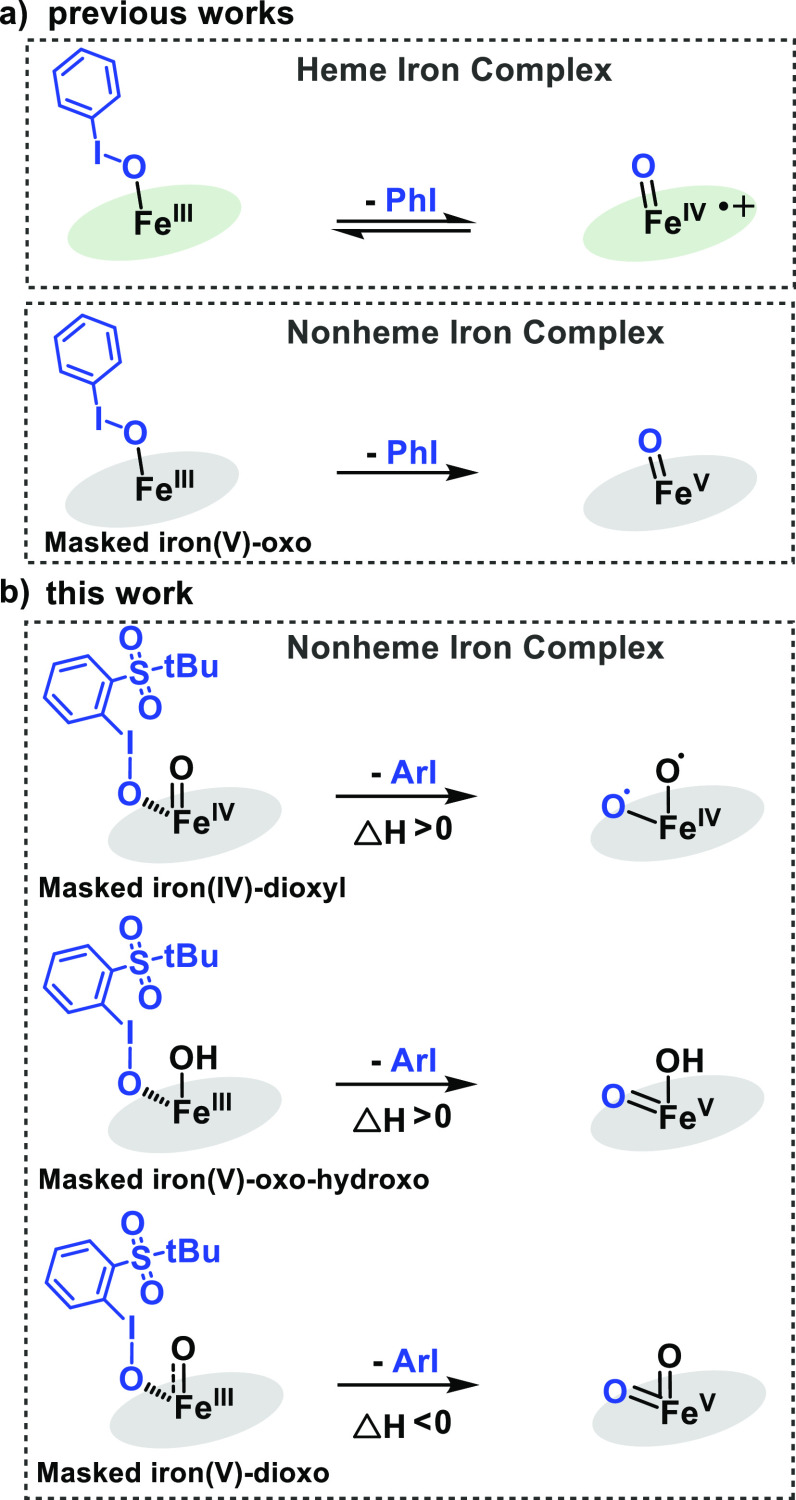
Previous studies on masked species of the type [(L)Fe^III^(PhIO)]^*n*+^ and the masked species (L)Fe^*n*^-OIAr species investigated in this work.

Most of the synthetic nonheme iron(IV)–oxo complexes are prepared from the oxidation of their iron(II) precursors with oxygen-atom transfer reactants such as H_2_O_2_^10,11^ or iodosylarenes^12^ (ArIO, the ligand depicted in blue in Figure 1). The ArIO oxidant can also act as a ligand^13,14^ and form “masked” iron–oxo complexes.^15−24^ For example, Nam et al. have reported that Fe^III^–OIAr is the active oxidant in the catalytic olefin epoxidation reactions (rather than Fe^IV^=O).^25^ Additionally, the Fe^III^–iodosylarene complexes are much more reactive than the analogous Fe^IV^=O species in olefin epoxidation. The formation of iron–iodosylarene can thus be an interesting way to form masked reactive species that could be generated during the reaction by the interaction with the second reactant. However, the mechanism of the unmasking of the Fe=O bond and what is the reactivity of the complexes after unmasking is unknown. So far, this reactivity was difficult to study in solution due to the short lifetime of all species involved. Here, we present unimolecular reactivity studies of masked and unmasked iron–oxo complexes.

We have previously compared the reactivity of [Fe^IV^O(TPA)(ArIO)]^2+^ (**1**^**2+**^, TPA = tris(2-pyridylmethyl)amine) with the iron(IV)–oxo complexes [Fe^IV^O(TPA)(MeCN)]^2+^, [Fe^IV^O(TPA)(OTf)]^+^, and [Fe^IV^O(TPA)(ArI)]^2+^. Complex **1**^**2+**^ stands out because of the Fe^IV^(O)(O-IAr) core. It has the weakest Fe^IV^=O bond, and it forms the strongest O–H bond after hydrogen atom transfer (HAT) among all the detected [(TPA)Fe^IV^O(X)]^2+/+^ species.^13^**1**^**2+**^ is the most reactive [(TPA)Fe^IV^O(X)]^2+/+^ complex for HAT, but it is the least reactive in oxygen atom transfer (OAT) reactions.^14^

**Figure 2:**
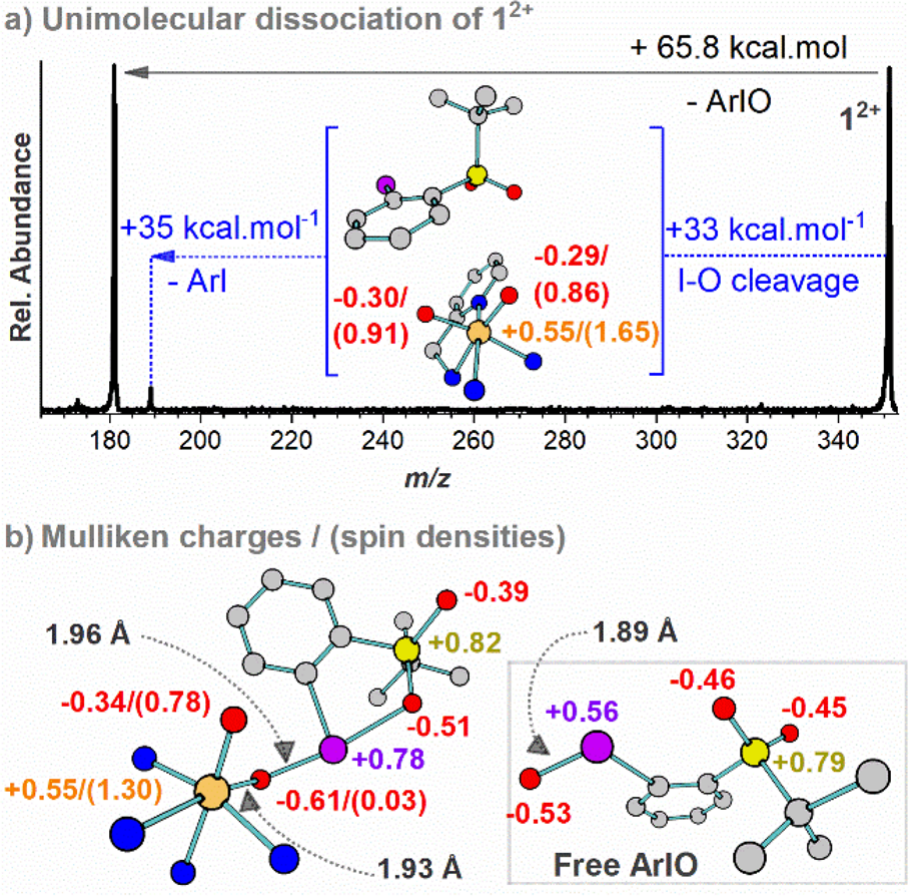
(a) Fragmentation spectra of the mass-selected ion **1**^**2+**^ upon collisions with xenon at 0.2 mTorr and 20 eV (*E*_lab_). The numbers next to the structures are spin densities (in between parentheses) and Mulliken charges (with a positive or negative sign next to it) at the atoms with the same color. Only the essential part of the TPA ligand is shown for clarity. (b) DFT properties of ^**3**^**1**^**2+**^ and at the free ArIO ligand. Atom color code: carbon, gray; iodine, purple; nitrogen, blue; oxygen, red; sulfur, yellow; iron, orange. DFT method: B3LYP-d3/def2svp.

The fragmentation of **1**^**2+**^ shows the expected elimination of ArIO (Figure 2a).^26^ However, the small competing channel corresponds to the elimination of ArI resulting in the formation of the iron(VI) dioxo complex. The peculiar effect of the ArIO ligand on the reaction selectivity of **1**^**2+**^ and the possibility to form iron(VI) dioxo complexes compelled us to study the properties and reactivity of **1**^**2+**^ in detail. We have used a combination of gas-phase ion–molecule reactions^27^ and DFT calculations to study the properties of **1**^**2+**^ and explore its reactivity toward the oxygenation of hydrocarbons.

## Experimental Section

### Chemical Synthesis

The chemicals 2-(*tert*-butylsulfonyl)iodosylbenzene (ArIO),^51^ [(TPA)Fe(OTf)_2_],^52^ [(TQA)Fe(OTf)_2_],^53^ and 1,4-cyclohexadiene-*d*_6_^54,55^ were prepared according to the published procedures. In detail, ArIO was obtained by the oxidation of 2-(*tert-*butylsulfonyl)iodobenzene with hydrogen peroxide in acetic anhydride at 32 °C during 24 h. The solvent was then removed under a reduced pressure which resulted in a white powder consisting of 2-(*tert-*butylsulfonyl)(diacetoxyiodo)benzene. The 2-(*tert-*butylsulfonyl)(diacetoxyiodo)benzene was converted to ArIO by a slow addition of the aqueous solution of NaOH (2 mM) over 1 h in the ice bath. A yellow precipitate corresponding to ArIO was collected by filtration, washed with H_2_O/diethyl ether, and dried under reduced pressure. We note that if the NaOH solution is added fast, ArIO disproportionates to ArI and ArIO_2_. The latter exhibits as a white solid precipitate. We have always carefully done the reaction to avoid this disproportionation. However, in order to exclude the possible involvement of ArIO_2_ in our experiments, we have also intentionally prepared ArIO_2_ and tested the unimolecular and bimolecular behavior of [(L)Fe(ArIO_2_)]^2+^ (see the Supporting Information). ArI^18^O was prepared by the addition of 50 μL of H_2_^18^O to a 1 mM solution of ArIO in dry acetonitrile, followed by sonication for 15 min. Incorporation of ^18^O was ensured by ESI(+)-MS. The remaining chemicals were commercially available.

### Generation of the Ions for the Gas-Phase Studies

The ions [(L)FeO(ArIO)]^2+^ (L = TPA or TQA) were generated by the mixing of the acetonitrile solutions of the iron triflate precursors [(L)Fe(OTf)_2_] (0.5 mM) with ArIO (1 mM) in a flow reactor directly connected to the electrospray source of the TSQ mass spectrometer, as previously described (Figure S1 and refs (13) and^14^). For the generation of [(TQA)FeO(ArIO)]^2+^, reaction times were kept short (∼3 s) to avoid complex decomposition. The mixed labeled complex [(TPA)Fe^18^O(ArI^16^O)]^2+^ was generated with the use of a flow reactor containing three lines and two mixing-Ts, as depicted in Figure S1a. The iron(II) precursor [(TPA)Fe(OTf)_2_] was oxidized by 1.1 equiv of ArI^18^O, and it led to the detection of [(TPA)Fe^18^O(ArI^18^O)]^2+^ (*m*/*z* 353 in Figure S1c). A third line containing a solution with ArI^16^O was then added to the flow reactor, and it led to detection of ^**16/18**^**1**^**2+**^ at *m*/*z* 352 as the major ion (Figure S1d).

### ESI(+)-MS Conditions

Typical ionization conditions were as follows: spray voltage (4 kV), capillary temperature (100 °C), sheath gas pressure (50 psi), no auxiliary gas, capillary voltage (5 V), and tube lens (60 V). All of the reactivity experiments were performed without prior thermalization of the generated ions because of their high reactivity. Such thermalization (pretrapping conditions) or capillary temperatures above 100 °C led to a larger fraction of decomposed iron–oxo species.^13^

### Gas-Phase Reactivity

All of the mass spectrometry experiments were performed in a TSQ mass spectrometer that has a quadrupole–octapole–quadrupole geometry and is equipped with an electrospray source. Collision-induced dissociation (CID) experiments were performed for ions that were transferred to the gas-phase via electrospray ionization, mass-selected by the first quadrupole, and accelerated to promote their thermal activation by collisions with xenon gas. The bimolecular reactivity experiments were performed for mass-selected ions at the zero-collision energy conditions (Figure S3). The kinetic energy distributions of the mass-selected ions were measured by retarding potential analysis and were almost identical for all investigated ions. The reactant gases (alkenes) were introduced from a test tube containing the corresponding sample and were degassed by freeze-evacuation-thaw cycles prior to the measurements. The neutral reactant pressure was 0.2 mTorr for all hydrocarbons. The exact pressure of TEMPO in the collision cell was impossible to measure (below the detection limit). The test tube with solid TEMPO must have been heated in order to get a sufficient gas pressure in the collision cell. The presence of TEMPO was detected by observing the bimolecular reactivity.

### DFT Calculations

Theoretical calculations were carried out with Density Functional Theory (DFT) using the Gaussian 16 package. The unrestricted B3LYP-D3 functional^56,57^ with the def2svp basis set with an effective core potential at the iodine atom was employed for all optimizations and frequency calculations. All calculations were performed in the gas phase. The stationary points were ascertained by vibrational frequency analysis with no imaginary frequencies at the minima (intermediates) and one imaginary frequency at the maxima (transition states).

## Results

### Structure of [(TPA)Fe^IV^O(ArIO)]^2+^ (1^2+^) and Its Dissociation to [(TPA)Fe^VI^(O)_2_]^2+^

First, we discuss the structure of gaseous **1**^**2+**^. We have spectroscopically characterized the structure of **1**^**2+**^ in our previous publication.^13^ The ions have a Fe=O bond characterized by the 834 cm^–1^ stretching frequency, and the ArIO ligand is coordinated by the iodosyl group (Figure S9). The complex has a triplet ground state (^**3**^**1**^**2+**^), the quintet and the singlet states lie 13.0 and 123.1 kJ mol^–1^, respectively, higher in energy. The ArIO ligand binds to the [Fe^IV^O(TPA)]^2+^ complex with exceptionally large binding energy via the oxygen atom of the iodosyl (IO) group (BDE_ArIO_ = 275.5 kJ mol^–1^ in the gas phase). The sulfone group of the ArIO molecule assists the binding by increasing the electronic density at the oxygen atom that binds to the iron center (Figure 2b). The sulfone assistance increases the binding energy by 36.0 kJ mol^–1^ in comparison to the binding of PhIO, where such assistance cannot exist (BDE_PhIO_ = 239.5 kJ mol^–1^ in the gas phase).

The I–O bond breaking in **1**^**2+**^ requires 139.4 kJ mol^–1^. The so formed free ArI molecule interacts with one of the pyridine rings of the TPA ligand by π-stacking. The following ArI dissociation requires an additional 146.1 kJ mol^–1^. The generated Fe^VI^-dioxo species has a quintet ground state that is better described as an Fe^IV^–dioxyl complex with two unpaired electrons at the iron center and one unpaired electron at each oxygen atom. The triplet spin state of the [(TPA)Fe^VI^(O)_2_]^2+^ complex lies 33.1 kJ mol^–1^ higher in energy than the quintet state, and it has a double oxo character with the iron center in the oxidation state +6. We expect that this highly reactive complex will probably self-oxidase in the gas phase.^28^ Most likely, one of the pyridine rings of the TPA ligand could be hydroxylated or transformed to an *N*-oxide.^30^

Our studies show that the unimolecular dissociation of **1**^**2+**^ via the direct I–O bond cleavage is highly endothermic.^31^ Next, we test the reactivity of **1**^**2+**^ in bimolecular reactions. We will show that unmasking the Fe=O bond of **1**^**2+**^ is exothermically achieved in two ways: (a) OAT reactivity with alkenes and (b) electron transfer reactions.

We note in passing that the alternative structure of the detected ions could correspond to [(TPA)Fe^II^(ArIO_2_)]^2+^. The ArIO_2_ molecule can be formed in a disproportionative degradation of ArIO (see the Experimental Section and the Supporting Information). We have prepared ArIO_2_ by the disproportionation of ArIO and generated the ions with *m*/*z* 351 using the ArIO_2_ oxidant. First, the general speciation of the complexes prepared by oxidation of [(TPA)Fe^II^(TfO)_2_] with ArIO_2_ is almost identical to that prepared in oxidation with ArIO (Figure S2). This attests to the fact that ArIO_2_ can also serve as an oxygen-atom transfer reagent and can generate iron(IV)oxo complexes along with ArIO that can react further. Second, the collision-induced dissociation patterns of the ions with *m*/*z* 351 prepared by oxidation with ArIO or ArIO_2_ are very similar. However, minor differences indicate that the ions prepared by the latter oxidant contain the [(TPA)Fe^II^(ArIO_2_)]^2+^ complexes (Figure S2). Namely, the ions eliminate ArIO_2_ in a larger abundance while the elimination of ArI (the double oxygen atom transfer) has almost disappeared. We assume that the ions represent a mixture of [(TPA)Fe^II^(ArIO_2_)]^2+^ and [(TPA)Fe^IV^(O)(ArIO)]^2+^. For the present study, we cannot exclude that the [(TPA)Fe^II^(ArIO_2_)]^2+^ ions contributed to the signal of the investigated ions with *m*/*z* 351. Nevertheless, we consider it rather unlikely. In order to obtain a reasonable yield of ArIO_2_ we must have sonicated the ArIO solution at room temperature for 3 h and the obtained ArIO_2_ reactant was barely soluble in acetonitrile (see the Supporting Information). If [(TPA)Fe^II^(ArIO_2_)]^2+^ contributed to the ions investigated here, then the reaction pathways would be similar. The reactivity would be preceded by the rearrangement of the iron(II) complex to the [(TPA)Fe^IV^(O)(ArIO)]^2+^ reactant as it likely happens during CID of [(TPA)Fe^II^(ArIO_2_)]^2+^ (Figure S2).^29^

### Gas-Phase Reactivity of 1^2+^ with Alkenes

**Figure 3:**
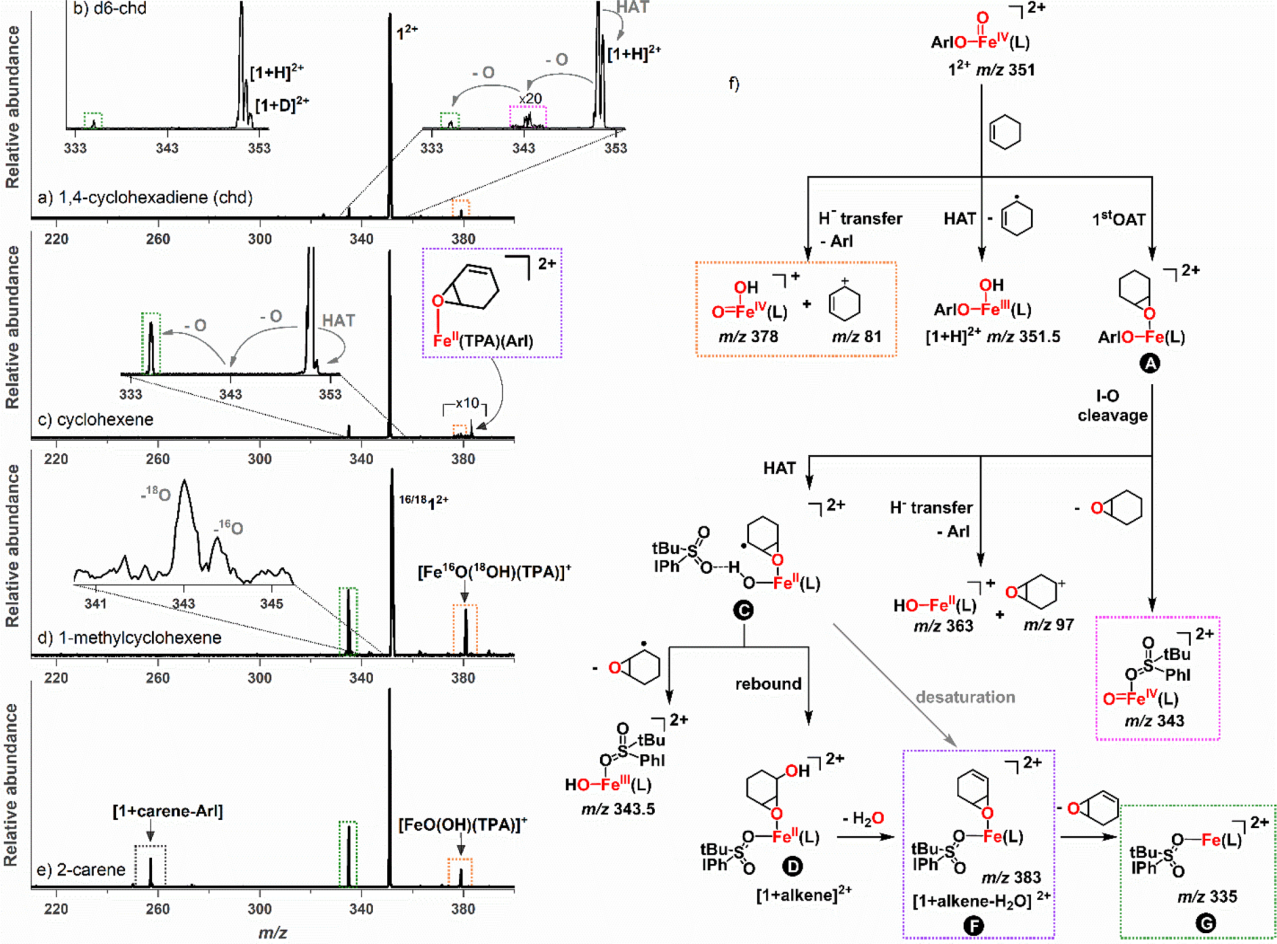
Gas-phase reactivity of **1**^**2+**^ with alkenes. (Left) ESI(+)-MS/MS spectra for the gas-phase collisions of **1**^**2+**^ (a) with 1,4-cyclohexadiene (the pink box contains the peaks of *m/*z 343 [(TPA)FeO(ArI)]^2+^ and *m/*z 343.5 [(TPA)FeOH(ArI)]^2+^ and their signal intensity was amplified by 20), (b) with 1,4-cyclohexadiene-*d*_6_ and (c) with cyclohexene. (d) Reactivity of the mixed labeled [(TPA)Fe^18^O(ArI^16^O)]^2+^ with 1-methylcyclohexene. (e) Reactivity of **1**^**2+**^ with 2-carene. The spectra were recorded at zero-collision energy and with ∼0.2 mTorr of the alkene pressure. (f) Scheme of the gas-phase reactivity of **1**^**2+**^ with cyclohexene. Some of the intermediates depicted in the PES of Figure 4 were omitted in Figure 3f for the sake of simplicity.

Iron(IV)oxo complexes react via HAT (detected as a hydrogen atom addition to the precursor ions) and OAT (detected as an oxygen atom loss from the precursor ions) with alkenes in the gas phase.^32^ The reaction of gaseous **1**^**2+**^ with 1,4-cyclohexadiene (chd) has a large selectivity for HAT over OAT (Figure 3a). Interestingly, we observe a small reaction channel that consists of two consecutive oxygen atom losses (see the pink and green boxes in Figure 3).

**Scheme 1:**
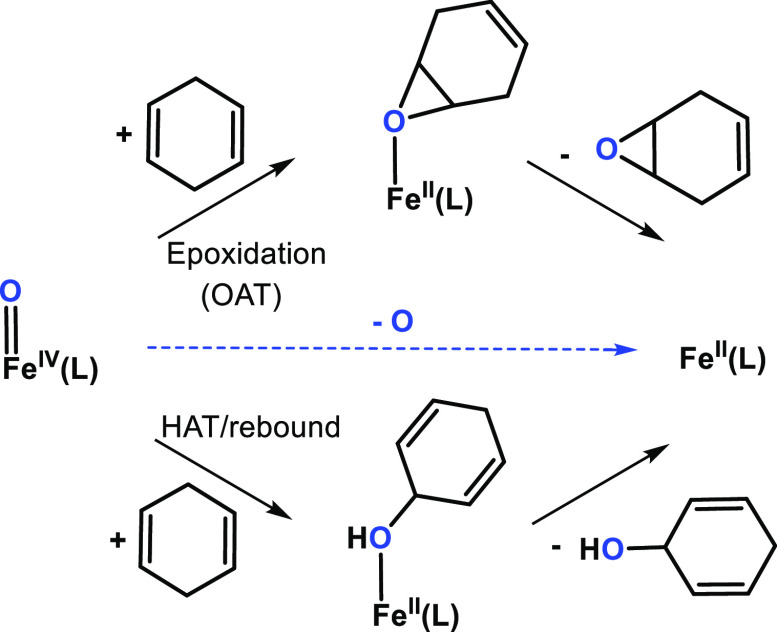
Two Possible Scenarios That Explain an Oxygen Atom Loss in the Gas Phase: (a) Alkene Epoxidation (OAT) or (b) Hydrogen Atom Transfer (HAT) Followed by the Rebound

A loss of the oxygen atom in the gas phase can be a result of (a) OAT to an alkene followed by the epoxide elimination from the reaction complex or (b) HAT followed by the radical rebound and the alcohol elimination from the reaction complex (Scheme 1).^33,34^ The rebound pathway after the initial HAT in the gas phase is usually observed only in the reactivity of naked or coordinatively unsaturated metal oxide cations.^35−39^ For fully coordinated systems as described here, the initial HAT reaction is followed by dissociation of the reaction complex which is barrierless and entropically favored.^40^ The mechanism of the double oxygen atom loss therefore probably starts via alkene epoxidation (simple OAT).

The reaction of **1**^**2+**^ with the partially deuterated 1,4-cyclohexadiene-*d*_6_ (d6-chd, Figure 3b) exhibits a kinetic isotope effect (KIE) of 3.07 ± 0.13 for the HAT channel. This KIE is not translated to the double oxygen atom loss channel (Figures 3a and S5), meaning that the initial HAT and the double oxygen atom loss channels are not consecutive reaction pathways. Therefore, we can exclude the rebound mechanisms and assume that OAT corresponds to the epoxidation reaction.

The reactions of **1**^**2+**^ with alkenes containing only one double bond (Figure 3c–e) also lead to HAT and formal double OAT. The fact that the alkenes have only one double bond excludes the possibility that we observed a double epoxidation reaction. Instead, the reactivity must correspond to OAT followed by HAT and the radical rebound within the complex. The branching ratio between HAT and the formal double OAT substantially decreased for 1-methylcyclohexene having stronger C–H bonds in comparison with 1,4-cyclohexadiene. This is yet another indication that the formal double OAT starts with the epoxidation of the double bond. We excluded the possibility of consecutive OAT with two molecules of alkene by measuring the reactivity dependence on the neutral reactant pressure (see figure S6).

Next, we tested which oxygen of **1**^**2+**^, the Fe=O or Fe–O–IAr, is transferred to the substrate in the initial OAT reaction using isotope labeling. The mixed labeled species ^**16/18**^**1**^**2+**^ was prepared by the oxidation of [(TPA)Fe(OTf)_2_] with ArI^18^O leading to [(TPA)Fe^18^O(ArI^18^O)]^2+^. Then we exchanged the labeled cis-ligand by ArI^16^O. The CID of ^**16/18**^**1**^**2+**^ shows a preferential loss of ArI^16^O (Figure S1e), meaning that ^**16/18**^**1**^**2+**^ consists mostly of [(TPA)Fe^18^O(ArI^16^O)]^2+^. The reaction of ^**16/18**^**1**^**2+**^ with 1-methylcyclohexene reveals that the first OAT occurs as the ^18^O loss, hence from the Fe=O group, and not from the Fe–O–IAr group (Figure 3c).

**Figure 4:**
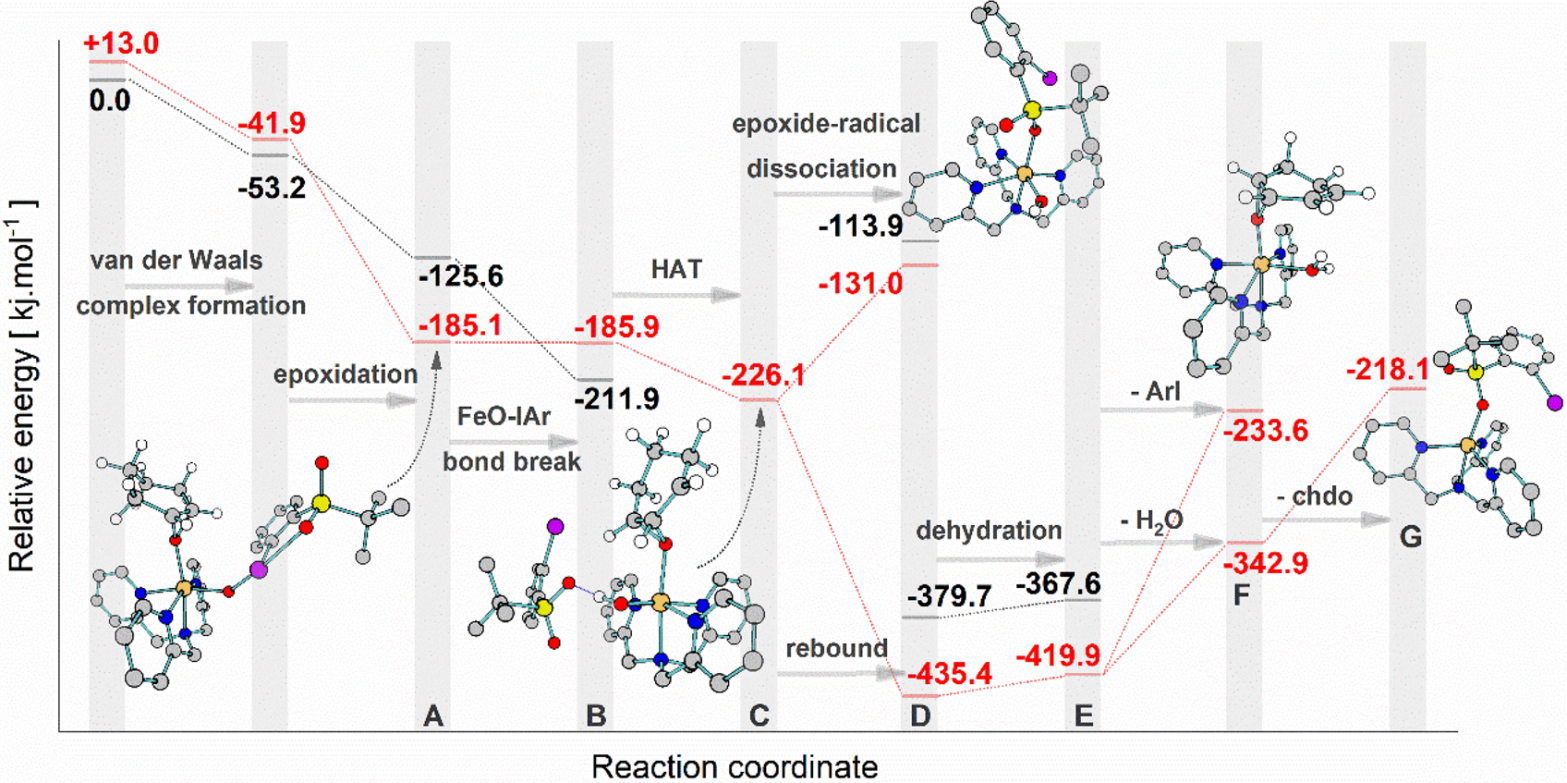
Potential energy surface for the gas-phase reaction of [(TPA)Fe^IV^O(ArIO)]^2+^ (**1**^**2+**^) with cyclohexene in the quintet (red) and triplet (black) spin states calculated at the B3LYP-d3/def2svp level of theory. chdo stands for 1,3-cyclohexadiene oxide.

The mechanism of the formal double oxygen atom transfer is rationalized based on the observed reaction products (see Figure 3f) and based on the DFT calculations (Figure 4). We have also explored alternative reaction pathways (see Figure S8). The letters under the structures of Figure 3f are used for guidance through the PES of Figure 4.

### DFT Rationalization of the Reaction Paths

The energy profile for the reaction between **1**^**2+**^ and cyclohexene is evidence of the remarkable reactivity of **1**^**2+**^, which can promote a full 4e^–^ oxidation of alkenes by a series of cascade events.

**1**^**2+**^ can initially react with cyclohexene (che) via OAT or HAT. Both reaction channels occur with a spin crossover from *S* = 1 to *S* = 2 surface, and the computed barriers are 8.8 kJ mol^–1^ and 11.3 kJ mol^–1^, respectively. The smaller energy barrier for OAT is consistent with the experimental selectivity in favor of the formal double oxygen atom transfer reactivity.

The initial HAT is exothermic by 53.6 kJ mol^–1^ (in comparison to the energy of the free reactants) and leads to the detection of [**1**+H]^2+^ (see the inset in Figure 3a,c). The HAT reaction can be also associated with subsequent electron transfer leading to the formation of two cations [**1**+H]^+^ + [che-H]^+^ (formal hydride transfer). The formation of two cations in the gas-phase is highly exothermic (so-called Coulomb explosion^41,42^); therefore, [**1**+H]^+^ further fragments by elimination of ArI to form [Fe^IV^O(OH)(TPA)]^+^ (orange boxes in Figure 3f).

The initial OAT is exothermic by 185.1 kJ mol^–1^, and it generates epoxide bounded to iron(II)-O-IAr (structure A in Figure 4 and Figure 3e). After OAT, the iron has oxidation state + II and the I–O bond breaking becomes exothermic, regenerating the iron(IV)-oxo at the cis position (B). The cis-iron(IV)-oxo promotes intramolecular HAT of the coordinated epoxide to form [(TPA)Fe^III^OH(epoxide-H)(ArI)]^2+^ (C). The intramolecular HAT is endothermic by 14.2 kJ mol^–1^ and it is stabilized by H-bonding interactions of the oxygen atom from the sulfone of ArI and the proton of Fe^III^OH.

The [(TPA)Fe^III^OH(epoxide-H)(ArI)]^2+^ complex is stabilized by the coordination of the epoxide to the iron center and therefore has a sufficient lifetime to allow a further radical reactivity leading to the formal double oxygen atom transfer. Two reaction paths are possible. In the first reaction path, Fe^III^OH abstracts another hydrogen atom from the C–H bond adjacent to the carbon radical to generate [(TPA)Fe^II^(H_2_O)(chdo)(ArI)]^2+^ (E, chdo is 1,3-cyclohexadiene oxide). This ion releases the internal energy by the elimination of H_2_O leading to the detected peak F at *m*/*z* 383 (Figure 3c). The complex F can further eliminate 1,3-cyclohexadiene leading to [(TPA)Fe^II^(ArI)]^2+^ (G) corresponding to the products of the formal double oxygen atom transfer. The second reaction path involves the rebound between Fe^III^OH and [epoxide-H] followed by the dehydration of the hydroxy-epoxide product D. This path also leads to complex F that can further produce G.

**Scheme 2:**
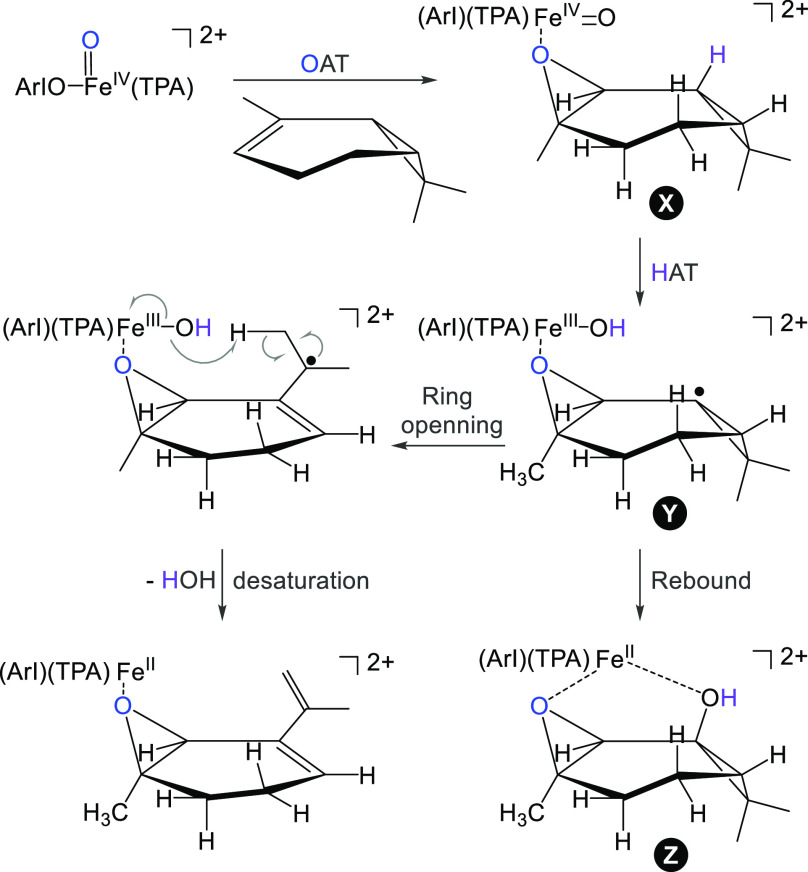
Possible Scenarios for the C–H Activation That Proceeds after the Initial OAT in the Reaction between Gaseous **1**^**2+**^ and 2-Carene

To test which scenario is correct, we investigated reaction with 2-carene. The initial OAT to this molecule leads to an epoxide complex (X in Scheme 2) that neighbors with a tertiary C–H bond and should thus preferentially react in the following HAT reaction. The so formed intermediate Y can react along the radical-rebound pathway and the product Z cannot easily lose H_2_O (Scheme 2). Only if the rebound does not proceed, the three-member ring should open and enable the desaturation pathway. Confirming the rebound scenario, we detected the product of the rebound mechanism that eliminated ArI instead of H_2_O (i.e., [**1**+carene-ArI]^2+^ in Figure 3e).

### Unmasking of the Fe=O Bonds

**Figure 5:**
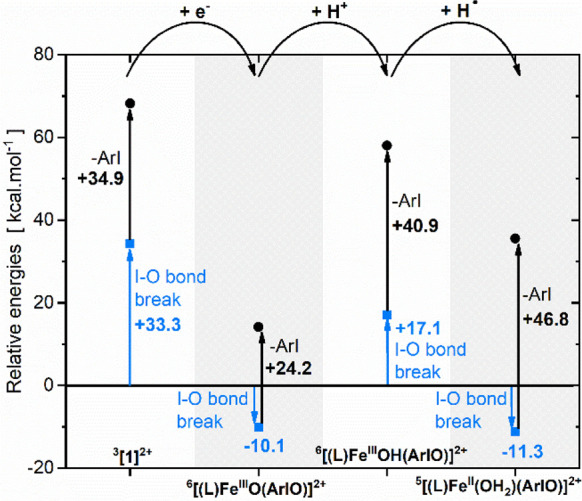
Energy diagram for ArI dissociation from the complexes ^a^[(L)Fe^b+^(ArIO)(X)]^c+^, where L is the TPA ligand, “a” is the spin multiplicity, “b” is the iron oxidation state, “c” is the complex charge, and X is an additional ligand depicted in the diagram.

The coordination of ArIO to [(TPA)FeO]^2+^ generates **1**^**2+**^, a 4e^–^ oxidant that is able to transfer both of its oxygen atoms to an alkene. The reason for this distinct reactivity is that the first OAT from **1**^**2+**^ generates [(TPA)Fe^II^(ArIO]^2+^ which undergoes an exothermic I–O bond cleavage and regenerates the Fe^IV^O moiety. The I–O bond cleavage does not happen if the reaction starts with HAT to produce [(TPA)Fe^III^(OH)(ArIO]^2+^. The unmasking of this complex to produce the iron(V)–oxo–hydroxo complex is endothermic (Figure 5).

**Figure 6:**
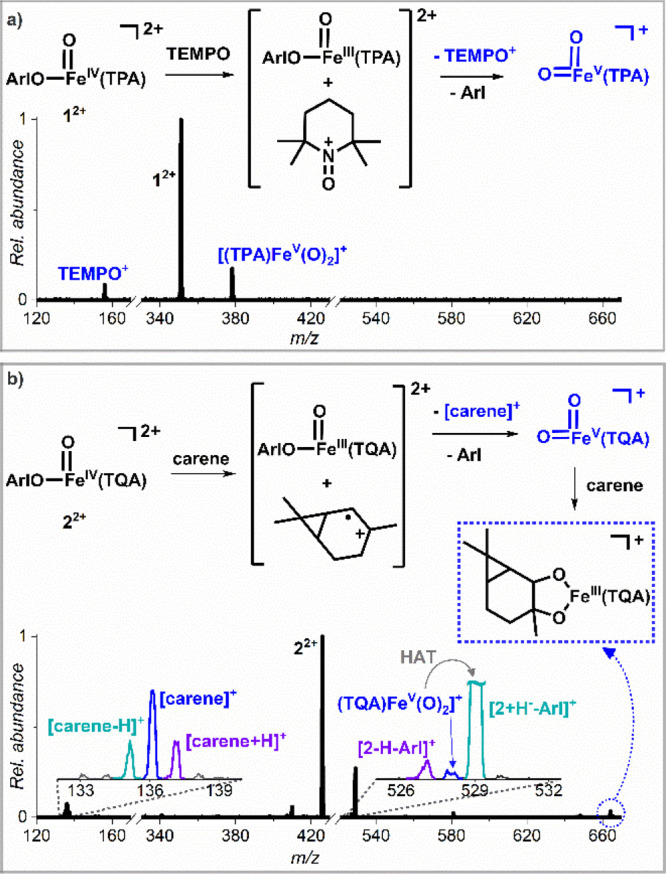
(a) Gas-phase reactivity of the mass-selected ion **1**^**2+**^ in collisions with TEMPO at zero-collision energy. (b) Gas-phase reactivity of the mass-selected ion **2**^**2+**^ in collisions with 2-carene (0.24 mTorr) at zero-collision energy. The main reactivity channels of **2**^**2+**^ in collisions with 2-carene are hydride transfer, double OAT, and electron transfer. The reactivity channels associated with the electron transfer from 2-carene to **2**^**2+**^ are highlighted in blue. For the full interpretation of the mass spectrum, see Figure S5.

We have also tested the unmasking chemistry for the iron(III) complex [(TPA)Fe^III^(O)(ArIO]^+^ (**1**^+^) formed by electron transfer to **1**^**2+**^ with TEMPO (2,2,6,6-tetramethylpiperidine-1-oxyl). TEMPO has a low ionization energy and therefore can transfer electron to **1**^**2+**^ (in contrast to the studied hydrocarbons). Theoretically, the I–O bond cleavage in **1**^+^ to form the Fe^V^=O complex is exothermic (Figure 5). Indeed, we can detect [(TPA)Fe^V^(O)_2_]^+^ after **1**^**2+**^ was reduced by TEMPO (Figure 6a).

We then tested the reactivity of the analogous complex [(TQA)FeO(ArIO)]^2+^ (**2**^**2+**^). The TQA (TQA = tris(2- quinolylmethyl)amine) ligand provides the same complex geometry but with a weaker ligand field than that of the TPA ligand.^43^ Therefore, **2**^**2+**^ has a larger electron affinity than **1**^**2+**^ and can transfer an electron with hydrocarbons such as 2-carene. The gas-phase collision between **2**^**2+**^ and 2-carene leads to the electron transfer reaction with the detection of [carene]^+^ and [(TQA)Fe^V^(O)_2_]^+^ (Figure 6b). The [(TQA)Fe^V^(O)_2_]^+^ species then reacts with a second molecule of 2-carene via HAT (Figures S5 and S7), or by formation of an adduct^44^ [(TQA)Fe^III^(carene+2O)]^+^.

The distinct reactivities of **1**^**2+**^ and **2**^**2+**^ show that the tuning of the thermochemistry of the FeO-IAr bond allows one to trigger new reaction pathways by the access of different masked iron–oxo intermediates. The enthalpy of the protonation of Fe^V^–dioxo species and the difference in the properties and the reactivity of Fe^v^-oxohydroxo and Fe^V^–dioxo species are still unknown. Gas-phase studies will be particularly useful on that topic because such species are too reactive and were so far never detected in solution.^45−48^

## Conclusions

The ArIO molecule is used as an oxygen-to-metal transfer reactant. It oxidizes Fe^II^ species to Fe^IV^=O. In addition, ArIO strongly binds to higher oxidation state iron complexes (Fe^n^), generating masked Fe^n+2^O species.

The I–O bond cleavage in gaseous **1**^**2+**^ generates [(TPA)Fe^VI^(O)_2_]^2+^, but this step is highly endothermic and achieved only under collision-induced dissociation conditions. Thermal unmasking of the Fe=O bond of **1**^**2+**^ is achieved by OAT reactivity that reduces the iron center to the oxidation state +II or by the one-electron reduction of **1**^**2+**^ to the iron(III) complex.

We demonstrate that the tuning of the thermochemistry of the I–O bond of masked iron–oxo species could be a promising strategy in oxidation chemistry. It allows access to highly reactive unmasked intermediates that can promote oxidative transformations via new and distinct reaction pathways. So far, most of the efforts on synthetic iron–oxo complexes have focused on understanding the ligand effects on their properties and reactivity. Our studies show that the choice of the oxidant used to oxidize the Fe^II^ precursor plays a key role. The tuning of the thermochemistry of the I–O bond of the ArIO oxidant by derivatization of the ArI precursor is a strategy that should be explored to induce I–O bond cleavage and allow new reaction pathways by access to even more reactive intermediates.

## Supporting Information Available

Additional experimental details, details about ion generation, details about gas-phase reactivity experiments–including kinetic energy resolution of the ions and pressure dependence of the reactivity, additional DFT calculations, and *XYZ* coordinates of the optimized structures (PDF)

## Dedication

This article is dedicated to Prof. Peter B. Armentrout. We would like to congratulate him on the award in honor of his contribution to gas-phase ion chemistry.
