# Supplementary material for: Unmasking the Iron–Oxo Bond of the [(Ligand)Fe-OIAr]2+/+ Complexes
Source: J Am Soc Mass Spectrom. 2022 Aug 3;33(9):1636–43. doi: 10.1021/jasms.2c00094 (PMC9460779; doi:10.1021/jasms.2c00094)
Supplement: Supplementary file 1 — js2c00094_si_001.pdf [file js2c00094_si_001.pdf]

SUPPORTING INFORMATION

for

Unmasking the iron-oxo bond of the $[(Ligand)Fe\text{-}OIAr]^{2+/+}$ complexes

Guilherme L. Tripodi and Jana Roithová*

Department of spectroscopy and Catalysis, Institute for Molecules and Materials, Radboud University, Heyendaalseweg 135, 6525 AJ Nijmegen, The Netherlands, E-mail: J.Roithova@science.ru.nl

Chemical Synthesis. The chemicals 2-(tert-butylsulfonyl)iodosylbenzene[S1] (2-(tert-$BuSO_2)C_6H_4IO)_2$, $[(TPA)Fe(OTf)_2]$[S2], $[(TQA)Fe(OTf)_2]$[S3] and 1,4-cyclohexadiene-[D6][S4,S5] were prepared according to published procedures. Additional chemicals were commercially available.

Generation of the ions for the gas-phase studies. The ions $[(L)FeO(ArIO)]^{2+}$ (L = TPA or TQA) were generated by the mixing of the acetonitrile solutions of the iron triflate precursors $[(L)Fe(OTf)_2]$ (0.5 mM) with ArIO (1 mM) in a flow reactor directly connected to the electrospray source of the TSQ mass spectrometer, as previously described (Figure S1 and references 13 and 14). For the generation of $[(TQA)FeO(ArIO)]^{2+}$, reaction times were kept short (~3 seconds) to avoid complex decomposition. The mixed labeled complex $[(TPA)Fe^{18}O(ArI^{16}O)]^{2+}$ was generated with the use of a flow reactor containing 3 lines and 2 mixing-Ts, as depicted in figure S1a. The iron(II) precursor $[(L)Fe(OTf)_2]$ was oxidized by 1.1 equivalents of $ArI^{18}O$ and it led to the detection of $[(TPA)Fe^{18}O(ArI^{18}O)]^{2+}$ (*m/z* 353 in Figure S1c). A third line containing a solution with $ArI^{16}O$ was then added to the flow-reactor and it led to detection of $^{16/18}\mathbf{1}^{2+}$ at *m/z* 352 as the major ion (Figure S1d). Upon CID (Figure S1e), $^{16/18}\mathbf{1}^{2+}$ fragments to $[(TPA)Fe(^{18}O)(^{16}O)]^{2+}$, $[(TPA)Fe^{18}O]^{2+}$ and $[(TPA)Fe^{16}O]^{2+}$. The preferential loss of the $ArI^{16}O$ ligand indicates that $^{16/18}\mathbf{1}^{2+}$ consists mostly of $[(TPA)Fe^{18}O(ArI^{16}O)]^{2+}$. Note that I-O bond cleavage requires lower energy than ArIO dissociation (Figure S1f) and for that reason, the ratio between the intensity of the fragments $[(TPA)Fe^{18}O]^{2+}$ and $[(TPA)Fe^{16}O]^{2+}$ is smaller than the ratio between $[(TPA)Fe^{18}O(ArI^{16}O)]^{2+}$ and $[(TPA)Fe^{16}O(ArI^{18}O)]^{2+}$ in the composition of the peak at *m/z* 352. Typical ionization conditions were: spray voltage (4 kV), capillary temperature (100°C), sheath gas pressure (50 psi), no auxiliary gas, capillary voltage (5 V) and tube lens (60 V). All the reactivity experiments were performed without the prior thermalization of the generated ions because of their high reactivity. Such thermalization (pre-trapping conditions) leads to a larger fraction of decomposed iron-oxo species.

The ions of *m/z* 351 that we study on the paper and assigned as $[(TPA)FeO(ArIO)]^{2+}$ could have an alternative formulation that consists of $[(TPA)Fe(ArIO_2)]^{2+}$. We have tested the preparation of the ions with m/z 351 using $ArIO_2$ oxidant prepared by disproportionation of ArIO (see Figure S2 for the comparison). The $ArIO_2$ was prepared by dissolving ArIO in MeCN (3mM) in the presence of a small amount of water (50 *uL* in 2 mL) and sonication at room temperature over 3 hours. This procedure led to the formation of a white precipitate that contains $ArIO_2$. The white precipitate was filtered, washed and partially re-dissolved in MeCN. The compound was barely soluble, therefore we must have sonicated the solution to achieve a dissolution, but we couldn't determine the exact concentration. We also cannot exclude that the white precipitate contained remaining traces of ArIO. This solution was used for the preparation of the ions $[(TPA)Fe^{II}(ArIO_2)]^{2+}$. Comparison of the source spectra obtained under the same ionization conditions using either ArIO or $ArIO_2$ oxidants reveals that the latter can also generate iron(IV)oxo complexes albeit slower than ArIO which is likely due to a smaller concentration of $ArIO_2$ (see above). The fact that the rate of the iron(IV)oxo complexes formation is slower is nicely illustrated by a larger abundance of iron(III) dimers that form, when the iron(II) complexes are not transformed to iron(IV)oxo complexes sufficiently fast (see Tripodi et al. Chem. Methods **2021**, *1*, 430-437, where we analyzed this process in a detail). Based on the CID patterns of the ions with *m/z* 351 we assume that the ions are a mixture of $[(TPA)Fe(ArIO_2)]^{2+}$ and $[(TPA)FeO(ArIO)]^{2+}$. The ions prepared using the $ArIO_2$ oxidant do not show any significant elimination of ArI (no double oxygen atom transfer upon collisional activation) and instead show a larger abundance of the $ArIO_2$ elimination and the ligand backbone fragmentation (Figure S2). For all ions (regardless of the oxidant used), the dominant fragmentation leads to a loss of ArIO suggesting the formation of the iron(IV)oxo core.

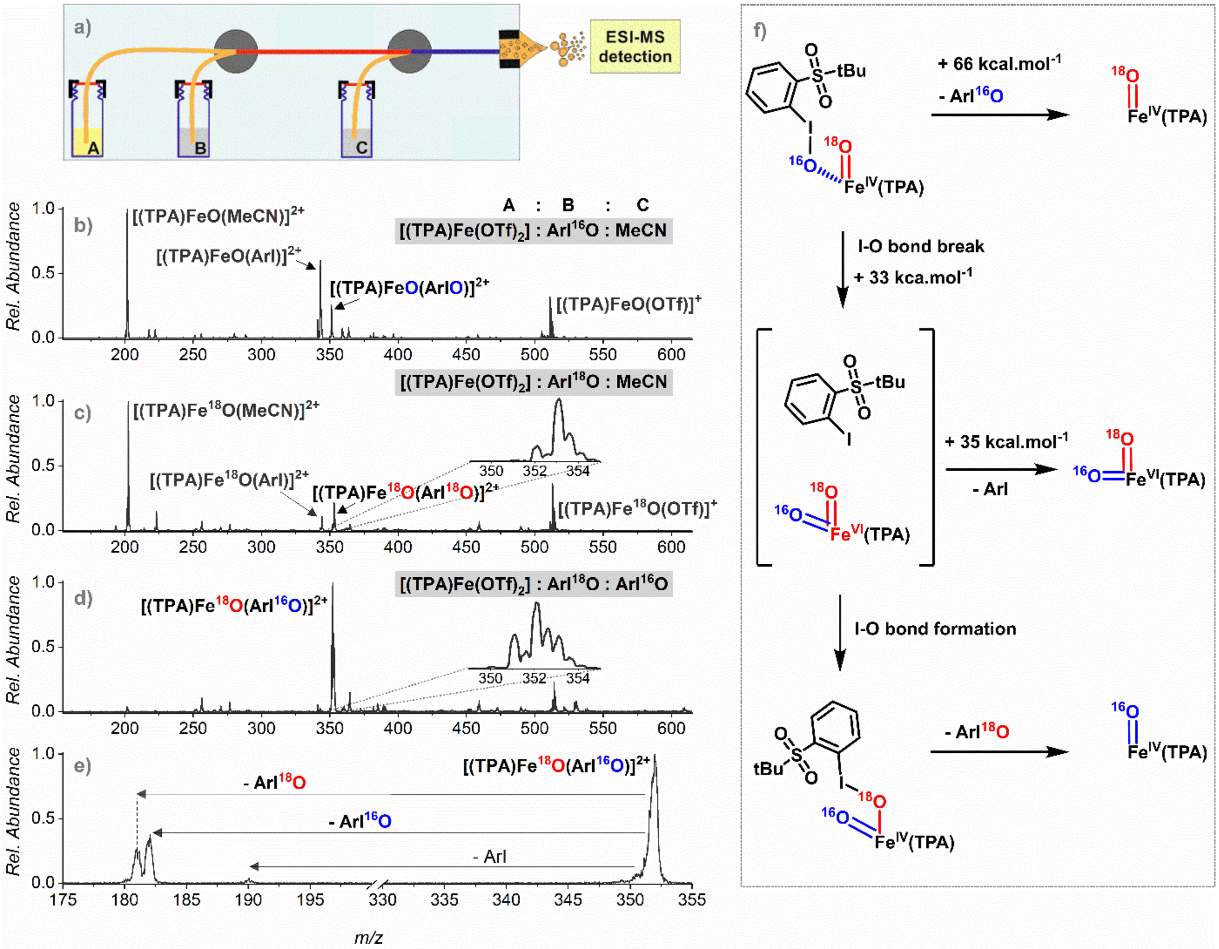

Figure S1. Generation of [(TPA)FeO(ArIO)]$^{2+}$ ions for the gas-phase reactivity studies. a) schematic representation of the flow-reactor used for the generation of the complexes. The composition of the vials A, B and C is depicted next to each spectrum. b) ESI(+)-MS for [(TPA)Fe(OTf)$_2$] (A) + ArI^{16}O (B). c) ESI(+)-MS for [(TPA)Fe(OTf)$_2$] (A) + ArI^{18}O (B). d) ESI(+)-MS for [(TPA)Fe(OTf)$_2$] (A) + ArI^{16}O (B) + ArI^{18}O (C). e) ESI(+)-MS/MS for the mass selected ions of *m/z* 352 ($^{16/18}$**1**$^{2+}$) uppon collisions with xenon gas at -35 eV (E$_{lab}$). f) Schematic representation of the fragmentation mechanism of $^{16/18}$**1**$^{2+}$ ions.

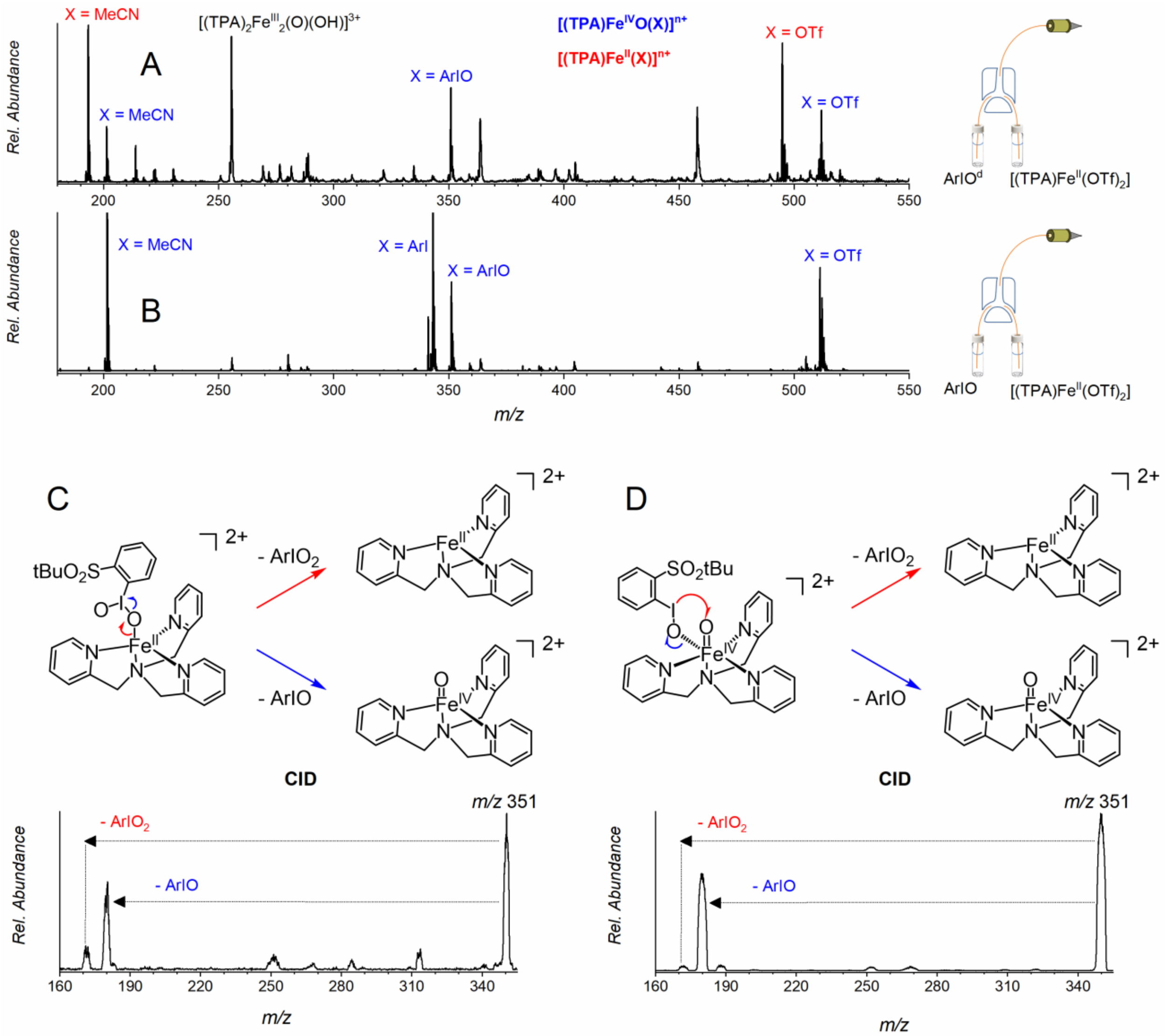

Figure S2. A) ESI(+)-MS spectra for the reaction between [((TPA)Fe(OTf)$_2$] and ArIO$_2$ formed by a disproportion of ArIO (see above, ArIO$_2$ – unknown concentration due to a poor solubility). B) ESI(+)-MS spectra for the reaction between [((TPA)Fe(OTf)$_2$] and a solution of freshly prepared ArIO (1.4 mM). C) and D) Fragmentation pattern of the mass selected ions of *m/z* 351 prepared from the conditions employed in A) and B), respectively (E_{lab}=15 *ev* and P_{Xenon}= 0.15 mTorr).

Gas-phase reactivity.

Unimolecular dissociation experiments (CIDs) were performed for mass-selected ions that were accelerated to promote their thermal activation by collisions with xenon gas. The bimolecular reactivity experiments were performed for mass-selected ions at zero-collision energy conditions (Figure S2). The reactant gases (alkenes) were introduced from a test tube containing the corresponding sample and were degassed by freeze-evacuation-thaw cycles prior to the measurements. The ion-molecule reactions were carried out at different pressures and different collision energies with a scan time of 3 seconds.

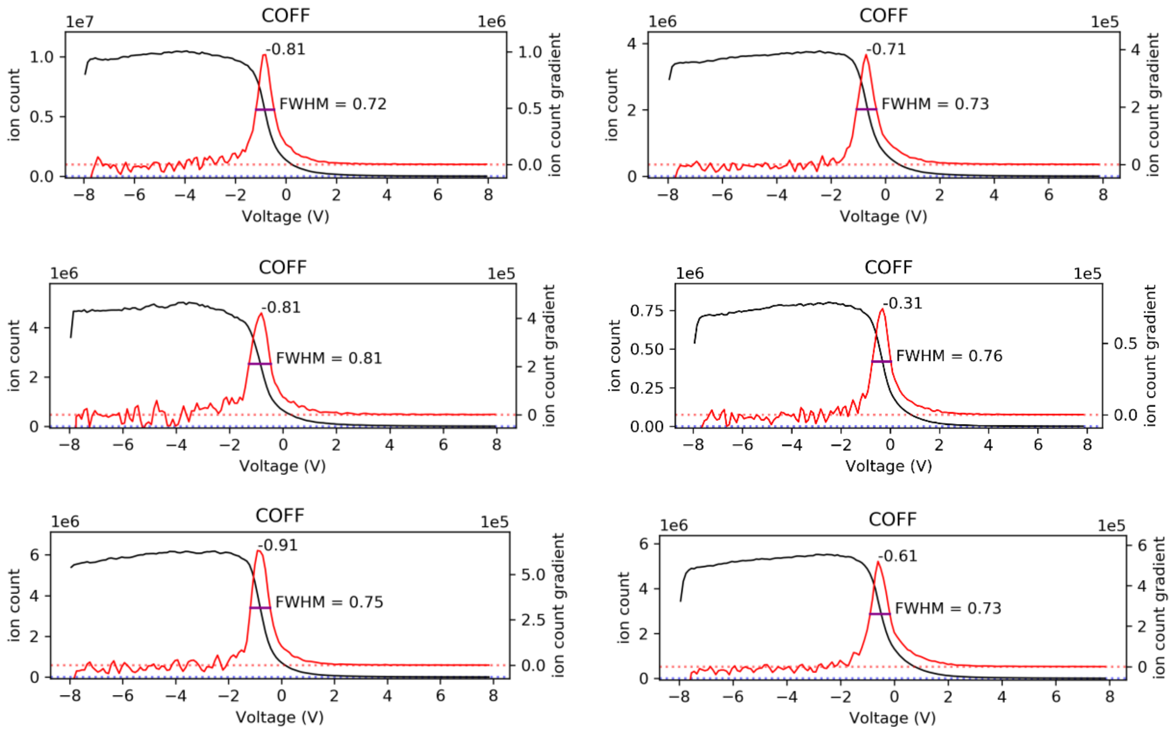

Figure S3. Typical ion kinetic distribution (red curve) measured at three different days by the stopping potential analysis for $[(TPA)FeO(ArIO)]^{2+}$ (left) and for $[(TQA)FeO(ArIO)]^{2+}$ (right). This type of measurement was repeated before each gas-phase reactivity measurement. The black curve is the ionic intensity as function of the applied collision offset. The full width at half maximum (FWHM) was tuned at ~0.75 *eV* for all the measurements.

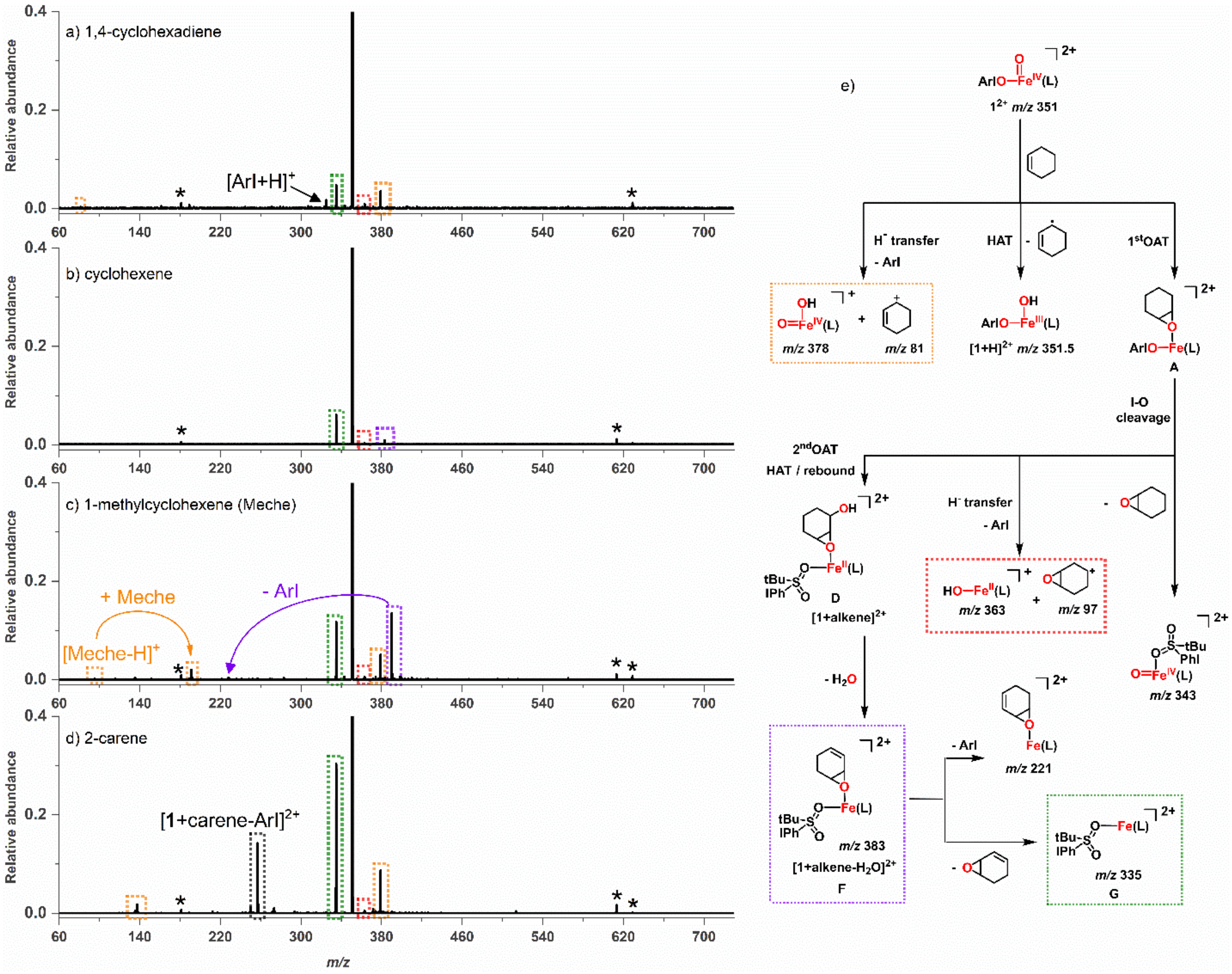

Figure S4. The gas-phase reactivity of 1^{2+} with alkenes. On the left, ESI(+)-MS/MS spectra for the gas-phase collisions of 1^{2+} with a) 1,4-cyclohexadiene, b) cyclohexene, c) 1-methylcyclohexene and e) 2-carene. The peaks with an asterisk (*) next to them are endothermic reaction channels produced via CID, as confirmed by their collision energy dependence (they are also detected in collisions with xenon gas). The peak at *m/z* 325 in Figure S3a corresponds to $[ArI+H]^+$. $[ArI+H]^+$ is formed after the initial HAT by proton abstraction from ArI and for that reason it is more pronounced for the reaction of 1^{2+} with strong hydrogen atom donors such as 1,4-cyclohexadiene. We also detect the corresponding fragment $[(TPA)Fe(O)_2]^+$ in small signal intensity. The peaks at *m/z* 335 and *m/z* 351.5 are not shown here because they are shown in Figure 3. The spectra were recorded at zero-collision energy and with ~0.2 mTorr of the alkene pressure. f) Scheme of the gas-phase reactivity of 1^{2+} with cyclohexene.

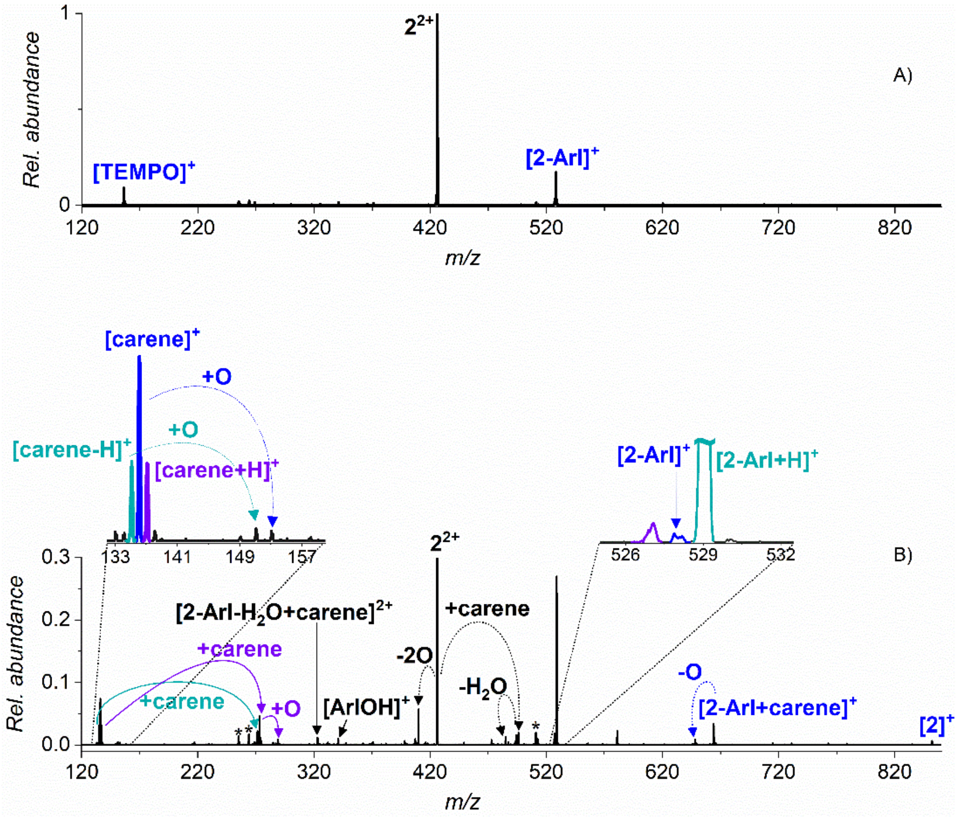

Figure S5. The gas-phase reaction between the mass-selected [(TQA)FeO(ArIO)]$^{2+}$ (**2**$^{2+}$) with A) TEMPO and B) 2-carene (0.24 mTorr) at zero-collision energy. The spectra reflects the high reactivity of **2**$^{2+}$, that is able to overcome the barriers of a variety of reaction channels at thermal conditions. The main observed reactivity patterns in the reaction of **2**$^{2+}$ with 2-carene are: electron transfer (blue), hydride transfer (green), proton transfer (purple), OAT/dOAT (loss of one/two oxygen atoms in analogy to **1**$^{2+}$, black) and a minor reaction channel associated to HAT. With exception of HAT and OAT/dOAT, all of the reaction channels lead to the formation of two monocharged daughter ions and are therefore very exothermic (Coulomb explosion). The peaks with an asterisk (*) next to them are endothermic reaction channels produced via CID, as confirmed by their collision energy dependence (they are also detected in collisions with xenon gas). Note that at larger pressures, the electron transfer channel prevails (see red curve of [carene]$^+$ in Figure S6) and it is accompanied by a larger increase in the cross-section of [(TQA)FeO(OH)]$^+$ (brown curve in Figure S6). This large increase is atributted to the HAT reactivity of the formed [(TQA)Fe^V(O)$_2$]$^+$. The same increase is not observed for the reaction of **1**$^{2+}$ with 2-carene because the ions [(TPA)FeO(OH)]$^+$ are only formed by the hydride transfer channel (**1**$^{2+}$ does not react via electron transfer with alkenes).

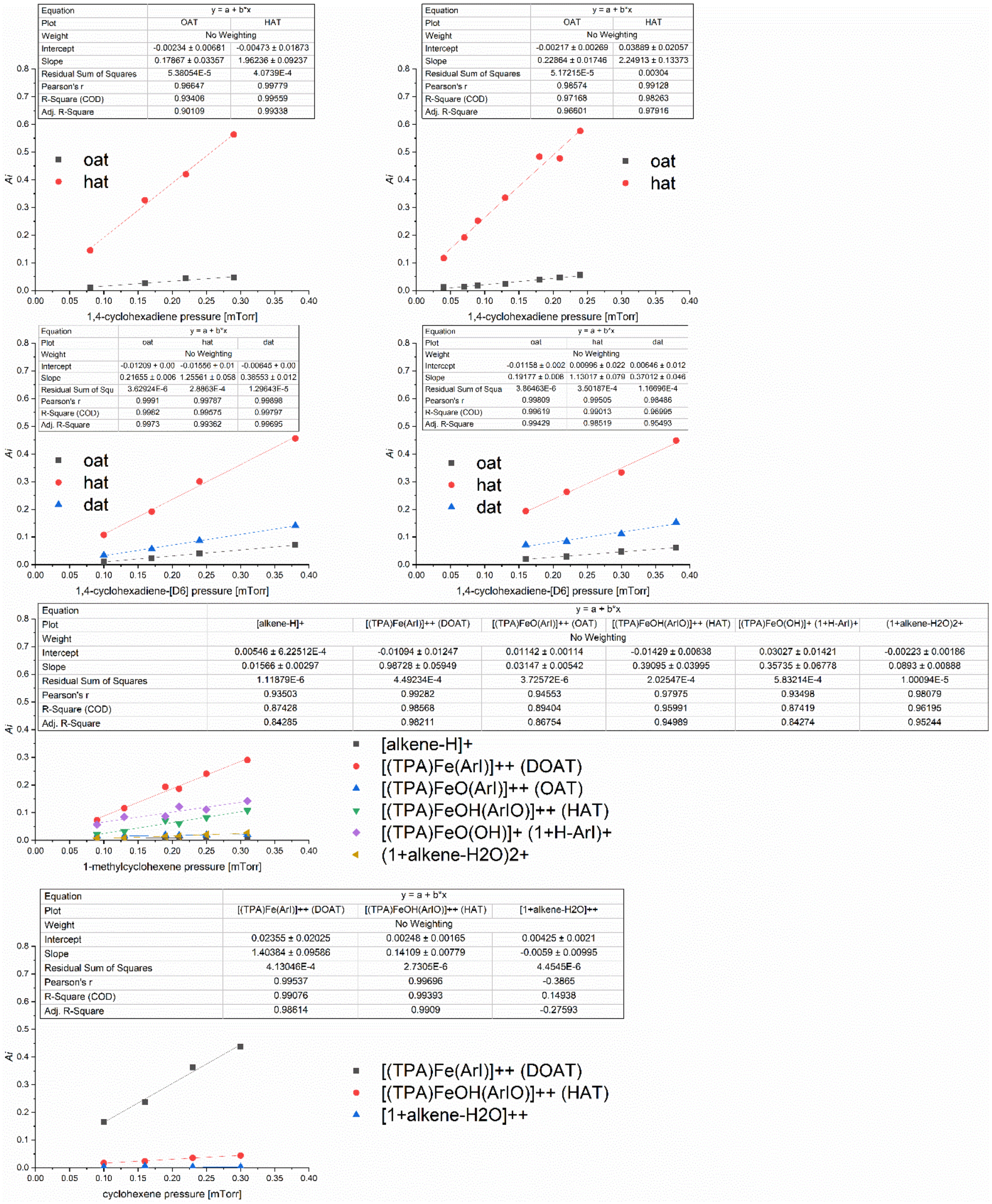

Figure S6. The pressure dependence reactivity of **1**$^{2+}$ in gas-phase collisions with alkenes. The relative cross-sections (A_i) for the fragmentation channel "i" was calculated according to the formula $A_i = -\ln(I_P/(I_P+I_F))*(I_i/I_F)$, where I_P, I_F and I_i are respectively, the intensity of the parent ions, the sum of the intensity of the daughter ions and the intensity of the daughter ion associated to the reaction channel i.

Equation	y = a + b*x									
Plot	I(135)	I (136)	I (410)	I(426p5)	I(485)	I(494)	I(528)	I(529)	I(664)	I(852)
Weight	No Weighting									
Intercept	-0.01914 ± 0.005	-0.02292 ± 0.00	-0.01513 ± 0.006	-0.00116 ± 5.15173	-0.00988 ± 0.00	-0.00462 ± 9.72491	-0.00232 ± 7.40899	-0.07963 ± 0.016	-0.0092 ± 3.49087	-0.00456 ± 0.001
Slope	0.25087 ± 0.045	0.35046 ± 0.040	0.27481 ± 0.049	0.03565 ± 0.00414	0.13017 ± 0.017	0.07966 ± 7.81965	0.04178 ± 0.00596	1.27825 ± 0.132	0.13092 ± 0.00281	0.06781 ± 0.009
Residual Sum of Squa	6.67623E-6	5.17665E-6	7.68246E-6	5.49109E-8	9.97071E-7	1.9567E-9	1.13572E-7	5.62011E-5	2.52128E-8	2.94411E-7
Pearson's r	0.98383	0.99348	0.98447	0.99332	0.99093	0.99995	0.98999	0.99467	0.99977	0.99014
R-Square (COD)	0.96791	0.987	0.96919	0.98668	0.98194	0.9999	0.98008	0.98937	0.99954	0.98038
Adj. R-Square	0.93583	0.974	0.93838	0.97336	0.96389	0.99981	0.96016	0.97873	0.99908	0.96077

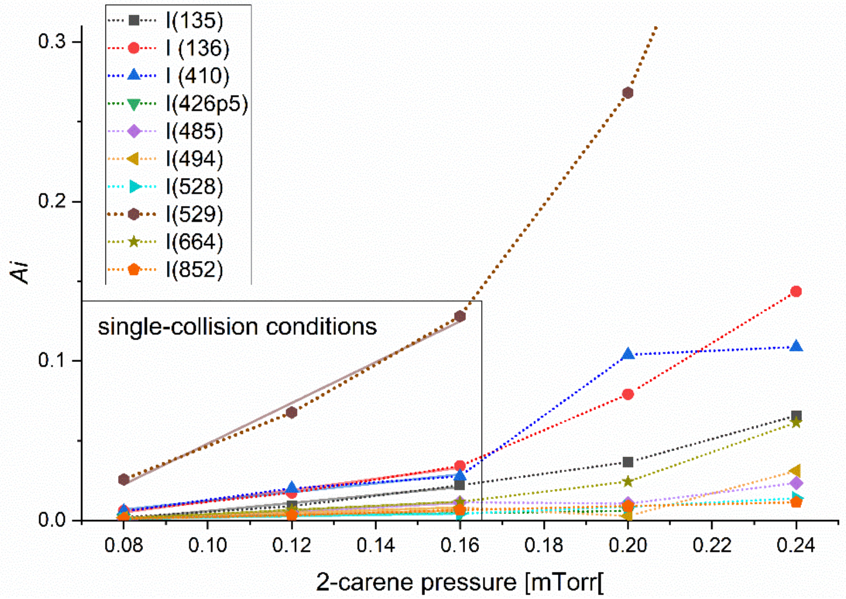

Figure S7. The pressure dependence reactivity of **2**$^{2+}$ in gas-phase collisions with 2-carene. The relative cross sections (A_i) for the fragmentation channel "i" was calculated according to the formula $A_i = -\ln(I_P/(I_P+I_F)) * (I_i/I_F)$, where I_P, I_F and I_i are respectively, the intensity of the parent ions, the sum of the intensity of the daughter ions and the intensity of the daughter ion associated to the reaction channel i. The linear regretion to obtain the relative rate constants (slope) was made by fitting of the points up to 0.17 mTorr of pressure, where collisions of **2**$^{2+}$ with more than one molecule of 2-carene can be neglected.

DFT Calculations

Theoretical calculations were carried out with Density Functional Theory (DFT) using the Gaussian 16 package. The unrestricted B3LYP-d3 functional[S6,S7] was employed for all optimizations and frequency calculations with def2svp level of theory for all atoms and with an effective core potential at the iodine atom. All calculations were performed in the gas phase. The stationary points were ascertained by vibrational frequency analysis with no imaginary frequencies at the minima (intermediates) and one imaginary frequency at the maxima (transition states).

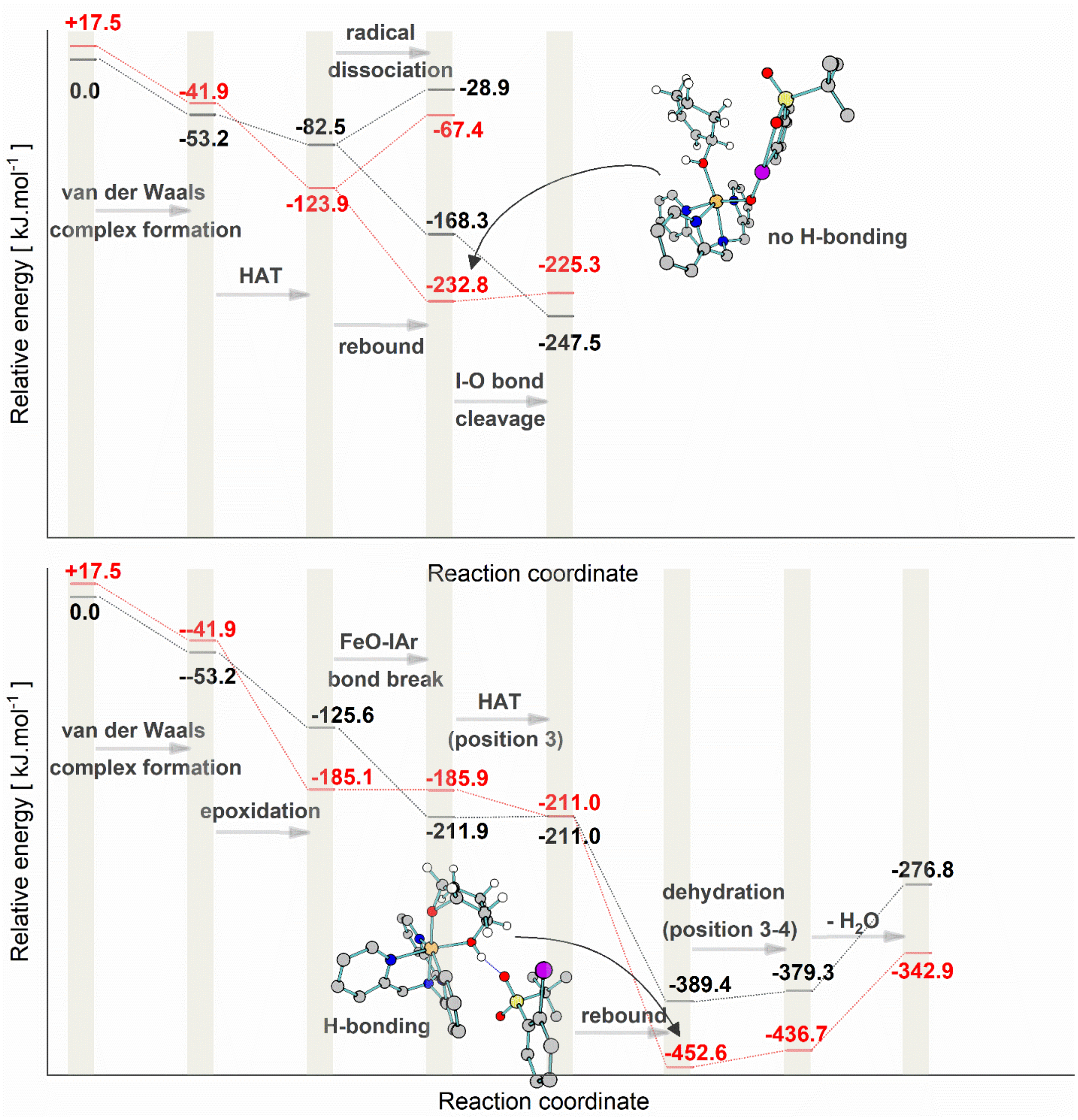

Figure S8. Potential energy surface for the gas phase reaction of [(TPA)FeIVO(ArIO)]$^{2+}$ (**1**$^{2+}$) with cyclohexene in the quintet (red) and triplet (black) spin states calculated at the B3LYP-d3/def2svp level of theory. The upper PES consists of the reaction pathway that starts via initial HAT, followed by rebound and I-O bond cleavage. The second PES consists of the pathway that starts via OAT (same as presented in Figure 4), but the intramolecular HAT step occurs at the position 3 of the bounded epoxide and not at the position 2. Note that, when OAT occurs prior to HAT/rebound, I-O bond break occurs and the ArI ligand interacts with the alcohol functionality via hydrogen bonding. Such hydrogen bonding interactions increase the exothermicity of the rebound step by 134 kJ.mol^{-1} (compare exothermicity of the rebound step on both PES).

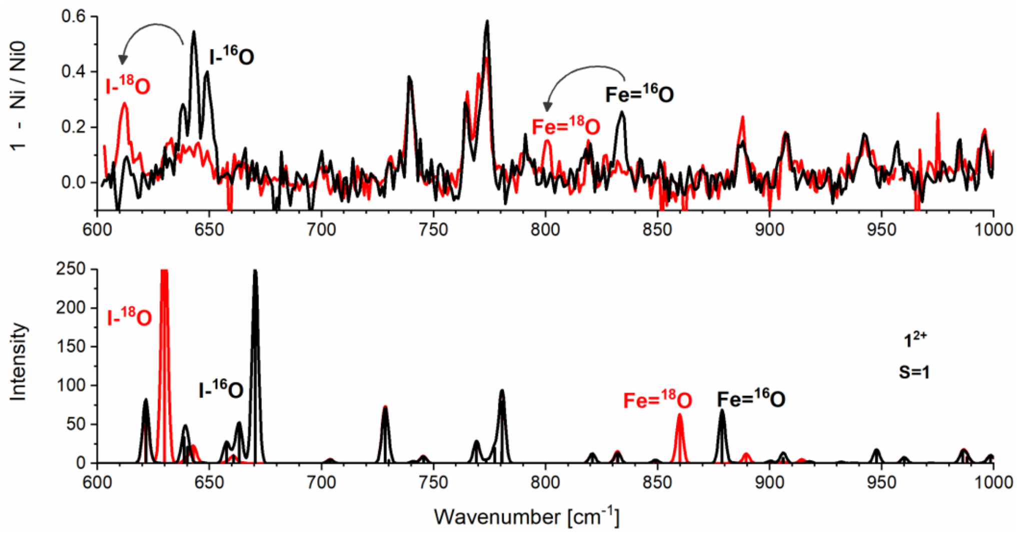

Figure S9. On the top, the helium tagging IRPD spectrum of the mass-selected ions of m/z 351 (black) and m/z 353 (red) generated by the oxidation of [(TPA)Fe(OTf)2] by ArI^{16}O and ArI^{18}O, respectively. Below, DFT predicted IR spectra for **1**$^{2+}$ (^{16}O-black) and **1**$^{2+}$ (^{18}O-red) calculated at the B3LYP-D3/Def2tzvp level of theory.

XYZ Coordinates

1^{2+} S=2

Fe	-1.699910000	0.062105000	0.239907000
N	-2.005784000	2.193596000	-0.314444000
N	-3.606656000	0.122436000	1.133199000
N	-2.162621000	-2.114382000	0.038843000
N	-2.991371000	-0.020469000	-1.568803000
O	-0.915298000	0.121385000	1.650769000
C	-2.513827000	1.106043000	-2.410300000
C	-2.421322000	2.369859000	-1.583655000
C	-2.706662000	3.642684000	-2.079803000
C	-2.546276000	4.746392000	-1.235095000
C	-2.116738000	4.546848000	0.080470000
C	-1.858028000	3.243847000	0.504867000
C	-4.420622000	0.145153000	-1.174775000
C	-4.670857000	0.050785000	0.313775000
C	-5.966240000	-0.049106000	0.827155000
C	-6.148562000	-0.061625000	2.211408000
C	-5.031368000	0.021831000	3.048982000
C	-3.770261000	0.106212000	2.468101000
C	-2.715929000	-1.342763000	-2.183970000
C	-2.711573000	-2.443245000	-1.145839000
C	-3.175802000	-3.735778000	-1.392257000
C	-3.047606000	-4.700529000	-0.386344000
C	-2.469423000	-4.344947000	0.835171000
C	-2.043265000	-3.027899000	1.010796000
H	-3.151384000	1.250514000	-3.298846000
H	-1.500094000	0.845780000	-2.750767000
H	-3.050652000	3.768429000	-3.108510000
H	-2.764770000	5.752951000	-1.599712000
H	-5.048390000	-0.588436000	-1.705494000
H	-4.767069000	1.136676000	-1.507499000
H	-6.820086000	-0.112416000	0.149020000
H	-7.153690000	-0.137949000	2.632900000
H	-1.705909000	-1.287866000	-2.616963000
H	-3.424842000	-1.562194000	-2.999865000
H	-3.626727000	-3.985745000	-2.354891000
H	-3.401524000	-5.720278000	-0.555476000
O	-0.200924000	-0.130318000	-0.924058000
I	1.524599000	-0.937999000	-0.389904000
C	2.398832000	0.931310000	0.225175000
C	3.704195000	0.958122000	0.714515000
C	4.219611000	2.147631000	1.243371000
H	5.217324000	2.158882000	1.685933000
C	3.436536000	3.304439000	1.214768000
H	3.836219000	4.236120000	1.620118000
C	2.144721000	3.266371000	0.679818000
H	1.536977000	4.173802000	0.655111000
C	1.604742000	2.069920000	0.191332000
H	0.587902000	2.016203000	-0.195998000
S	4.693513000	-0.547867000	0.765281000
O	3.689946000	-1.591849000	0.279072000
O	5.282214000	-0.706872000	2.090752000
C	6.031921000	-0.488589000	-0.544984000
C	6.670730000	-1.882586000	-0.463383000
H	7.486602000	-1.922576000	-1.201586000
H	5.949180000	-2.676232000	-0.703293000
H	7.098309000	-2.071945000	0.531436000
C	7.016186000	0.613448000	-0.148962000
H	7.392111000	0.470935000	0.874267000
H	7.875608000	0.563771000	-0.835184000
H	6.577150000	1.617153000	-0.246109000
C	5.356374000	-0.227877000	-1.890130000
H	4.837684000	0.743416000	-1.912335000
H	6.137558000	-0.200847000	-2.665398000
H	4.649725000	-1.027727000	-2.153467000
H	-1.522878000	3.016676000	1.520678000
H	-1.991961000	5.383701000	0.770047000
H	-2.850569000	0.156281000	3.055890000
H	-5.132368000	0.014580000	4.135558000
H	-2.354875000	-5.070514000	1.642467000
H	-1.596285000	-2.678810000	1.945742000

1^{2+} S=1

Fe	-1.805162000	0.061756000	0.136655000
N	-2.134702000	1.971232000	-0.311059000
N	-3.589496000	-0.032313000	1.047645000
N	-1.809464000	-1.923019000	0.119725000
N	-3.007600000	-0.205993000	-1.605840000

O	-0.983937000	0.287142000	1.515191000
C	-2.643709000	0.963866000	-2.447284000
C	-2.565951000	2.193264000	-1.572131000
C	-2.871035000	3.482612000	-2.006084000
C	-2.714649000	4.551721000	-1.118008000
C	-2.267043000	4.300892000	0.182211000
C	-1.988057000	2.986979000	0.552828000
C	-4.448188000	-0.197444000	-1.225409000
C	-4.667562000	-0.227130000	0.268601000
C	-5.938620000	-0.398541000	0.821744000
C	-6.085957000	-0.355328000	2.208680000
C	-4.956093000	-0.143261000	3.005278000
C	-3.719701000	0.009686000	2.386963000
C	-2.555776000	-1.510667000	-2.148411000
C	-2.273469000	-2.479006000	-1.022199000
C	-2.403638000	-3.862173000	-1.139351000
C	-2.032561000	-4.673153000	-0.061383000
C	-1.548218000	-4.079085000	1.107226000
C	-1.455424000	-2.689946000	1.163267000
H	-3.342616000	1.104427000	-3.288051000
H	-1.639725000	0.778127000	-2.853962000
H	-3.226996000	3.645479000	-3.025309000
H	-2.948723000	5.569970000	-1.437406000
H	-4.975726000	-1.039285000	-1.701397000
H	-4.918221000	0.716929000	-1.621213000
H	-6.799606000	-0.560076000	0.169441000
H	-7.070797000	-0.485947000	2.663162000
H	-1.609718000	-1.332537000	-2.679112000
H	-3.276064000	-1.930430000	-2.869320000
H	-2.787645000	-4.297726000	-2.064027000
H	-2.125445000	-5.759328000	-0.133739000
O	-0.292297000	0.086009000	-1.061682000
I	1.391985000	-0.775388000	-0.531730000
C	2.285005000	1.033394000	0.228013000
C	3.571667000	1.013230000	0.764168000
C	4.082235000	2.164323000	1.376650000
H	5.063509000	2.135963000	1.853851000
C	3.316293000	3.332802000	1.383705000
H	3.712981000	4.234715000	1.854135000
C	2.045776000	3.344692000	0.798518000
H	1.453256000	4.262501000	0.799805000
C	1.509610000	2.184863000	0.225517000
H	0.509909000	2.162635000	-0.206993000
S	4.547032000	-0.503129000	0.754592000
O	3.555903000	-1.510999000	0.188041000
O	5.105413000	-0.735193000	2.083495000
C	5.919070000	-0.371167000	-0.513740000
C	6.550532000	-1.770921000	-0.501071000
H	7.384999000	-1.771643000	-1.219273000
H	5.831558000	-2.544253000	-0.806197000
H	6.951154000	-2.022220000	0.491277000
C	6.898804000	0.698919000	-0.028921000
H	7.247005000	0.493583000	0.993419000
H	7.775379000	0.685161000	-0.694801000
H	6.466904000	1.709230000	-0.076680000
C	5.281287000	-0.026655000	-1.858762000
H	4.768413000	0.947690000	-1.836584000
H	6.082276000	0.040383000	-2.611040000
H	4.576528000	-0.804129000	-2.186477000
H	-1.632400000	2.713699000	1.548683000
H	-2.141840000	5.108221000	0.905879000
H	-2.794862000	0.167134000	2.944999000
H	-5.026690000	-0.100988000	4.093478000
H	-1.249140000	-4.678384000	1.968848000
H	-1.090720000	-2.154510000	2.042488000

Encounter complex S=2

Fe	-2.161526000	0.225043000	-0.243510000
N	-1.896896000	-1.710792000	0.775409000
N	-3.954347000	-0.654170000	-0.869979000
N	-3.038340000	2.177407000	-0.526101000
N	-3.341633000	0.495461000	1.564873000
O	-1.461244000	0.019862000	-1.687497000
C	-2.695557000	-0.342840000	2.602997000
C	-2.303447000	-1.698722000	2.058855000
C	-2.272567000	-2.854207000	2.840852000
C	-1.789520000	-4.038606000	2.272282000
C	-1.360211000	-4.032947000	0.942905000
C	-1.439453000	-2.841019000	0.221528000
C	-4.757640000	0.097324000	1.303028000

C	-4.945022000	-0.705376000	0.037481000
C	-6.122907000	-1.409339000	-0.220882000
C	-6.268907000	-2.053806000	-1.451620000
C	-5.233880000	-1.980379000	-2.389371000
C	-4.082750000	-1.272694000	-2.056231000
C	-3.233349000	1.948178000	1.865097000
C	-3.482621000	2.751143000	0.608763000
C	-4.081602000	4.010351000	0.595742000
C	-4.202386000	4.683935000	-0.625196000
C	-3.730684000	4.078207000	-1.793161000
C	-3.153310000	2.811913000	-1.700744000
H	-3.331933000	-0.437506000	3.498767000
H	-1.773262000	0.177199000	2.903430000
H	-2.618707000	-2.828562000	3.876328000
H	-1.755461000	-4.957490000	2.862584000
H	-5.363549000	1.012066000	1.204101000
H	-5.167703000	-0.448535000	2.167690000
H	-6.916050000	-1.445901000	0.529010000
H	-7.182849000	-2.608713000	-1.676437000
H	-2.198633000	2.134779000	2.189277000
H	-3.912251000	2.246176000	2.681692000
H	-4.446506000	4.457320000	1.522818000
H	-4.666841000	5.672170000	-0.663018000
O	-0.664396000	0.920208000	0.707872000
I	1.090854000	1.234218000	-0.144638000
C	2.101089000	-0.180875000	1.122012000
C	3.475499000	-0.375585000	0.993619000
C	4.104690000	-1.398124000	1.712114000
H	5.170053000	-1.586195000	1.567089000
C	3.351775000	-2.182036000	2.589372000
H	3.837776000	-2.980734000	3.153089000
C	1.980921000	-1.948566000	2.736283000
H	1.392685000	-2.561137000	3.423232000
C	1.340205000	-0.948707000	1.994265000
H	0.267393000	-0.782372000	2.069264000
S	4.434547000	0.609307000	-0.168419000
O	3.346227000	1.371303000	-0.912022000
O	5.335045000	-0.258750000	-0.926164000
C	5.428384000	1.897050000	0.757994000
C	6.076399000	2.724232000	-0.361683000
H	6.697389000	3.501788000	0.109366000
H	5.323274000	3.218776000	-0.991350000
H	6.725308000	2.103980000	-0.996078000
C	6.471135000	1.156972000	1.597739000
H	7.086546000	0.488197000	0.979333000
H	7.137003000	1.906709000	2.052085000
H	6.013301000	0.584483000	2.417922000
C	4.456838000	2.715951000	1.606640000
H	3.943616000	2.098718000	2.360589000
H	5.035177000	3.481374000	2.146500000
H	3.710578000	3.233768000	0.987442000
H	-1.121322000	-2.779922000	-0.821728000
H	-0.974997000	-4.934774000	0.463897000
H	-3.226438000	-1.185581000	-2.729337000
H	-5.311771000	-2.468397000	-3.362340000
H	-3.810906000	4.573902000	-2.762199000
H	-2.767624000	2.278089000	-2.573369000
C	1.339762000	-1.821399000	-2.051640000
C	1.856823000	-1.317710000	-3.374512000
C	3.365689000	-1.546484000	-3.539275000
C	3.768720000	-2.941232000	-3.046261000
C	3.429489000	-3.118000000	-1.559186000
C	2.045563000	-2.616119000	-1.231554000
H	0.330212000	-1.501555000	-1.774929000
H	1.611806000	-0.245083000	-3.480372000
H	1.298658000	-1.820701000	-4.187471000
H	3.656575000	-1.406906000	-4.591969000
H	3.916968000	-0.790988000	-2.956338000
H	4.843602000	-3.113568000	-3.209957000
H	3.231704000	-3.706322000	-3.634601000
H	3.522282000	-4.176375000	-1.260032000
H	4.167789000	-2.569503000	-0.945282000
H	1.609241000	-2.925506000	-0.273379000

Encounter complex S=1

Fe	-2.240512000	0.142857000	-0.141834000
N	-1.840990000	-1.545844000	0.816889000
N	-3.928403000	-0.716872000	-0.793578000
N	-2.921967000	1.942430000	-0.635024000
N	-3.434892000	0.520043000	1.578380000

O	-1.438077000	-0.159126000	-1.517032000
C	-2.772355000	-0.261347000	2.650527000
C	-2.238628000	-1.568328000	2.108528000
C	-2.067086000	-2.711393000	2.887735000
C	-1.454537000	-3.834492000	2.319827000
C	-1.034783000	-3.783339000	0.989087000
C	-1.254644000	-2.615521000	0.260369000
C	-4.836159000	0.085712000	1.315559000
C	-4.978966000	-0.716741000	0.045062000
C	-6.163846000	-1.381447000	-0.278149000
C	-6.255547000	-2.046551000	-1.501560000
C	-5.157330000	-2.030162000	-2.367241000
C	-4.004781000	-1.357350000	-1.975313000
C	-3.333164000	1.994025000	1.746990000
C	-3.435029000	2.655301000	0.392309000
C	-3.965740000	3.927018000	0.184314000
C	-3.945317000	4.467978000	-1.105729000
C	-3.403095000	3.718428000	-2.152730000
C	-2.901188000	2.447591000	-1.877386000
H	-3.437568000	-0.424831000	3.514053000
H	-1.911837000	0.329411000	2.996893000
H	-2.405844000	-2.719901000	3.925791000
H	-1.310850000	-4.740662000	2.913099000
H	-5.474127000	0.979226000	1.225250000
H	-5.229697000	-0.483689000	2.172578000
H	-7.002848000	-1.371162000	0.420978000
H	-7.173089000	-2.571966000	-1.776435000
H	-2.334025000	2.210899000	2.149552000
H	-4.087857000	2.383166000	2.449931000
H	-4.386779000	4.486440000	1.022120000
H	-4.354072000	5.464150000	-1.290435000
O	-0.793608000	1.000060000	0.823263000
I	0.928749000	1.295368000	-0.070060000
C	2.017671000	-0.051853000	1.209624000
C	3.383526000	-0.259023000	1.021527000
C	4.046851000	-1.245730000	1.758765000
H	5.103226000	-1.444846000	1.570292000
C	3.339455000	-1.980948000	2.713067000
H	3.853121000	-2.751172000	3.291694000
C	1.979567000	-1.731658000	2.921275000
H	1.428596000	-2.300959000	3.673253000
C	1.304243000	-0.767773000	2.162238000
H	0.239021000	-0.582964000	2.290416000
S	4.288077000	0.672299000	-0.226559000
O	3.175335000	1.416498000	-0.942735000
O	5.150084000	-0.232424000	-0.987019000
C	5.334740000	1.983166000	0.605215000
C	5.933868000	2.763892000	-0.573573000
H	6.580650000	3.554325000	-0.162453000
H	5.153967000	3.239312000	-1.185083000
H	6.548369000	2.115551000	-1.214163000
C	6.412957000	1.268072000	1.421255000
H	6.989705000	0.567522000	0.800735000
H	7.107875000	2.028408000	1.809717000
H	5.992713000	0.734997000	2.286784000
C	4.411306000	2.841844000	1.468250000
H	3.928424000	2.256670000	2.266543000
H	5.020223000	3.620272000	1.953167000
H	3.641018000	3.344123000	0.865935000
H	-0.949515000	-2.511696000	-0.782043000
H	-0.545031000	-4.632413000	0.509589000
H	-3.106338000	-1.314141000	-2.593586000
H	-5.186773000	-2.535829000	-3.333853000
H	-3.371158000	4.105444000	-3.172643000
H	-2.465038000	1.803745000	-2.644290000
C	1.539384000	-1.809192000	-2.093860000
C	2.145023000	-1.616205000	-3.460629000
C	3.617692000	-2.046017000	-3.516289000
C	3.833408000	-3.368742000	-2.771371000
C	3.444294000	-3.233120000	-1.292226000
C	2.123811000	-2.525285000	-1.119504000
H	0.569903000	-1.331304000	-1.917353000
H	2.038232000	-0.559135000	-3.762963000
H	1.546635000	-2.189065000	-4.195473000
H	3.946921000	-2.131211000	-4.563494000
H	4.240199000	-1.267693000	-3.046039000
H	4.881230000	-3.695831000	-2.856964000
H	3.217036000	-4.157782000	-3.238190000
H	3.405135000	-4.222976000	-0.804869000
H	4.226408000	-2.664541000	-0.755422000

H	1.634507000	-2.606679000	-0.141064000

TS HAT S=2

Fe	-1.944019000	0.163166000	0.051808000
N	-2.297344000	2.272759000	0.336140000
N	-3.896194000	-0.216977000	0.893574000
N	-2.315080000	-1.738015000	-0.943454000
N	-3.337261000	0.735763000	-1.663600000
O	-1.089739000	-0.284208000	1.431133000
C	-2.897823000	2.107453000	-2.001849000
C	-2.764036000	2.934499000	-0.741299000
C	-3.064526000	4.295785000	-0.673795000
C	-2.866759000	4.972623000	0.534220000
C	-2.384241000	4.270678000	1.643304000
C	-2.111951000	2.911175000	1.501055000
C	-4.749279000	0.694256000	-1.208562000
C	-4.964790000	-0.034096000	0.101962000
C	-6.240522000	-0.437786000	0.505760000
C	-6.398260000	-1.026085000	1.762374000
C	-5.277220000	-1.200341000	2.580366000
C	-4.037277000	-0.787082000	2.102308000
C	-3.035467000	-0.254266000	-2.718010000
C	-2.910579000	-1.651431000	-2.148521000
C	-3.318357000	-2.796790000	-2.833787000
C	-3.086215000	-4.049385000	-2.255429000
C	-2.462310000	-4.122012000	-1.006847000
C	-2.096958000	-2.932979000	-0.377185000
H	-3.570664000	2.595742000	-2.727998000
H	-1.899114000	2.027126000	-2.457593000
H	-3.450594000	4.816839000	-1.552513000
H	-3.097666000	6.037669000	0.610614000
H	-5.395737000	0.260597000	-1.989136000
H	-5.103332000	1.728553000	-1.065758000
H	-7.098026000	-0.288336000	-0.154402000
H	-7.386866000	-1.347521000	2.099220000
H	-2.056207000	0.014988000	-3.142159000
H	-3.778050000	-0.225945000	-3.534232000
H	-3.808151000	-2.710308000	-3.805569000
H	-3.395930000	-4.959718000	-2.774099000
O	-0.462081000	0.521053000	-1.213025000
I	1.266556000	-0.379142000	-1.087456000
C	2.188782000	1.151361000	0.113345000
C	3.542543000	1.085576000	0.440340000
C	4.089394000	2.023041000	1.323645000
H	5.137063000	1.941194000	1.619315000
C	3.276419000	3.038886000	1.832710000
H	3.698043000	3.773682000	2.521417000
C	1.928367000	3.109690000	1.466158000
H	1.295706000	3.907814000	1.861985000
C	1.367491000	2.157034000	0.605965000
H	0.313705000	2.182140000	0.331088000
S	4.575600000	-0.249774000	-0.187214000
O	3.553476000	-1.192741000	-0.794429000
O	5.439357000	-0.745135000	0.885893000
C	5.628538000	0.381713000	-1.598392000
C	6.353138000	-0.875636000	-2.098582000
H	7.011206000	-0.579173000	-2.930114000
H	5.647111000	-1.631244000	-2.470253000
H	6.975953000	-1.321363000	-1.309851000
C	6.602170000	1.410271000	-1.019864000
H	7.191030000	0.987299000	-0.193817000
H	7.300306000	1.706355000	-1.817844000
H	6.088390000	2.321082000	-0.677563000
C	4.695291000	0.977582000	-2.650941000
H	4.126542000	1.836840000	-2.261990000
H	5.310471000	1.342050000	-3.487913000
H	3.997529000	0.227281000	-3.049405000
H	-1.734803000	2.296689000	2.322594000
H	-2.229817000	4.764143000	2.604581000
H	-3.112738000	-0.908215000	2.671070000
H	-5.359866000	-1.655480000	3.568690000
H	-2.267924000	-5.081036000	-0.523254000
H	-1.617956000	-2.913516000	0.604636000
C	1.856316000	-0.753864000	3.217862000
C	0.582783000	-1.486863000	3.113563000
C	0.684830000	-2.848612000	2.427383000
C	1.985801000	-3.575358000	2.793553000
C	3.202258000	-2.718999000	2.418917000
C	3.049101000	-1.295773000	2.869068000
H	1.826286000	0.266784000	3.614565000

H	-0.149833000	-0.836089000	2.458266000
H	0.030376000	-1.499319000	4.071174000
H	-0.195083000	-3.465443000	2.669304000
H	0.672865000	-2.688409000	1.333097000
H	2.033918000	-4.554623000	2.291039000
H	1.999939000	-3.773516000	3.878940000
H	4.130804000	-3.136281000	2.841583000
H	3.362188000	-2.725860000	1.323347000
H	3.959526000	-0.696919000	2.952594000

TS HAT S=1

Fe	-1.989266000	0.103333000	-0.181432000
N	-2.431876000	2.040134000	-0.370535000
N	-3.627010000	-0.175465000	0.942506000
N	-1.913848000	-1.888460000	-0.372415000
N	-3.375166000	-0.050463000	-1.756204000
O	-0.942403000	0.412292000	1.179672000
C	-3.184662000	1.216735000	-2.510385000
C	-3.048048000	2.358319000	-1.528955000
C	-3.476197000	3.660731000	-1.782532000
C	-3.248035000	4.647067000	-0.817193000
C	-2.607322000	4.299774000	0.375149000
C	-2.214584000	2.975365000	0.564352000
C	-4.756390000	-0.172462000	-1.208963000
C	-4.784432000	-0.367845000	0.287604000
C	-5.964007000	-0.674736000	0.970151000
C	-5.935872000	-0.767469000	2.362026000
C	-4.728107000	-0.546488000	3.032058000
C	-3.591693000	-0.254469000	2.285167000
C	-2.935653000	-1.264786000	-2.486305000
C	-2.492058000	-2.333965000	-1.512443000
C	-2.612094000	-3.698859000	-1.774363000
C	-2.116198000	-4.616956000	-0.843383000
C	-1.526621000	-4.142140000	0.330639000
C	-1.451306000	-2.765415000	0.530971000
H	-3.996292000	1.393721000	-3.235108000
H	-2.237748000	1.131665000	-3.061557000
H	-3.980173000	3.898497000	-2.721448000
H	-3.575233000	5.674436000	-0.993280000
H	-5.297033000	-0.990788000	-1.710628000
H	-5.315328000	0.748014000	-1.441018000
H	-6.890941000	-0.832188000	0.414433000
H	-6.845730000	-1.005280000	2.917919000
H	-2.066249000	-0.980187000	-3.095148000
H	-3.717113000	-1.642808000	-3.165712000
H	-3.088790000	-4.037320000	-2.696570000
H	-2.198586000	-5.690171000	-1.030138000
O	-0.636793000	0.317994000	-1.546799000
I	1.091160000	-0.562187000	-1.294940000
C	2.027384000	1.121772000	-0.327403000
C	3.347722000	1.054185000	0.117248000
C	3.884832000	2.120325000	0.850393000
H	4.899183000	2.050641000	1.246751000
C	3.103916000	3.254383000	1.082276000
H	3.520485000	4.089520000	1.648967000
C	1.793855000	3.317238000	0.595864000
H	1.186466000	4.207107000	0.775962000
C	1.237217000	2.241398000	-0.104507000
H	0.207100000	2.253511000	-0.457033000
S	4.336800000	-0.426696000	-0.157710000
O	3.334926000	-1.376771000	-0.784100000
O	4.982053000	-0.818329000	1.098060000
C	5.632305000	-0.095515000	-1.468006000
C	6.290289000	-1.467214000	-1.676300000
H	7.079051000	-1.351556000	-2.435607000
H	5.569424000	-2.213470000	-2.038945000
H	6.755700000	-1.834854000	-0.750741000
C	6.618877000	0.928462000	-0.904302000
H	7.030272000	0.603052000	0.061894000
H	7.455053000	1.022608000	-1.614122000
H	6.166391000	1.924997000	-0.797472000
C	4.908628000	0.403979000	-2.717934000
H	4.378388000	1.352248000	-2.537965000
H	5.661640000	0.590712000	-3.498963000
H	4.200726000	-0.343010000	-3.104382000
H	-1.703584000	2.619311000	1.462239000
H	-2.419996000	5.040524000	1.154500000
H	-2.616343000	-0.068100000	2.734803000
H	-4.663828000	-0.600341000	4.120104000
H	-1.133985000	-4.823964000	1.086804000

H	-1.013611000	-2.335548000	1.429359000
C	0.658083000	0.353472000	3.794067000
C	0.348906000	-0.850987000	3.037822000
C	1.540755000	-1.660720000	2.541622000
C	2.718032000	-1.602024000	3.529767000
C	3.117422000	-0.148577000	3.813687000
C	1.930404000	0.723659000	4.098021000
H	-0.176783000	0.983051000	4.119017000
H	-0.298882000	-0.329431000	1.997599000
H	-0.498578000	-1.428125000	3.445791000
H	1.252208000	-2.704541000	2.341388000
H	1.891645000	-1.252776000	1.579004000
H	3.576264000	-2.153612000	3.118630000
H	2.427443000	-2.093401000	4.473640000
H	3.823241000	-0.096278000	4.659283000
H	3.676486000	0.254076000	2.949215000
H	2.099245000	1.667264000	4.627395000

TS OAT S=2

Fe	-1.778575000	0.147039000	-0.072299000
N	-2.106956000	2.280665000	0.164192000
N	-3.750790000	-0.100753000	0.801020000
N	-2.225996000	-1.751926000	-1.015586000
N	-3.191842000	0.715527000	-1.806266000
O	-0.906617000	-0.309047000	1.297351000
C	-2.740261000	2.076636000	-2.162901000
C	-2.595194000	2.921888000	-0.915387000
C	-2.906816000	4.281985000	-0.864367000
C	-2.698731000	4.979587000	0.329535000
C	-2.194150000	4.298725000	1.442108000
C	-1.911901000	2.939960000	1.315115000
C	-4.604923000	0.685217000	-1.360421000
C	-4.823770000	0.104649000	0.023304000
C	-6.116572000	-0.160105000	0.488634000
C	-6.285694000	-0.633809000	1.789768000
C	-5.157969000	-0.833059000	2.593987000
C	-3.905624000	-0.557482000	2.055650000
C	-2.889131000	-0.297115000	-2.837394000
C	-2.800410000	-1.681878000	-2.232566000
C	-3.229301000	-2.834534000	-2.892649000
C	-3.043402000	-4.076812000	-2.276905000
C	-2.444744000	-4.132144000	-1.015253000
C	-2.054494000	-2.936808000	-0.413417000
H	-3.409062000	2.562871000	-2.894383000
H	-1.743314000	1.981439000	-2.619093000
H	-3.309062000	4.785795000	-1.745653000
H	-2.938041000	6.043807000	0.392837000
H	-5.224544000	0.133977000	-2.087093000
H	-5.002485000	1.713376000	-1.352095000
H	-6.977597000	0.007289000	-0.162480000
H	-7.286460000	-0.846128000	2.173287000
H	-1.899013000	-0.053231000	-3.250380000
H	-3.618101000	-0.276060000	-3.666088000
H	-3.701440000	-2.760685000	-3.874501000
H	-3.370395000	-4.991984000	-2.776195000
O	-0.322215000	0.474929000	-1.395815000
I	1.407000000	-0.413738000	-1.250831000
C	2.274722000	1.086565000	0.028083000
C	3.571606000	0.952948000	0.522936000
C	4.045823000	1.858467000	1.479652000
H	5.038165000	1.718861000	1.912372000
C	3.231924000	2.921720000	1.880078000
H	3.600575000	3.634711000	2.620316000
C	1.951166000	3.068288000	1.336249000
H	1.319022000	3.904948000	1.642589000
C	1.454378000	2.139031000	0.414360000
H	0.445007000	2.214296000	0.011042000
S	4.615755000	-0.425482000	0.009684000
O	3.655379000	-1.269266000	-0.806660000
O	5.247587000	-1.014081000	1.191312000
C	5.923481000	0.183293000	-1.183340000
C	6.637748000	-1.104604000	-1.618416000
H	7.437992000	-0.825724000	-2.321236000
H	5.954004000	-1.795768000	-2.131209000
H	7.096409000	-1.619400000	-0.762247000
C	6.856102000	1.120045000	-0.413881000
H	7.265852000	0.634097000	0.483067000
H	7.698366000	1.379932000	-1.073407000
H	6.359233000	2.059716000	-0.131184000
C	5.208642000	0.877283000	-2.342145000

H	4.642066000	1.761287000	-2.009861000
H	5.970799000	1.224624000	-3.056544000
H	4.535790000	0.190681000	-2.875290000
H	-1.516619000	2.342609000	2.140810000
H	-2.030570000	4.808206000	2.393409000
H	-2.979818000	-0.700805000	2.614536000
H	-5.247121000	-1.200435000	3.617713000
H	-2.287566000	-5.081898000	-0.501163000
H	-1.593321000	-2.902089000	0.575926000
C	-0.071579000	-0.841861000	3.313922000
C	-0.531154000	-2.263267000	3.455634000
C	0.389692000	-3.257268000	2.736460000
C	1.860294000	-2.957841000	3.042902000
C	2.227170000	-1.546837000	2.567957000
C	1.211958000	-0.520412000	2.953462000
H	-0.724390000	-0.053770000	3.696216000
H	-1.572425000	-2.355347000	3.104123000
H	-0.568400000	-2.497699000	4.537227000
H	0.129194000	-4.287749000	3.022523000
H	0.233448000	-3.175205000	1.645842000
H	2.521427000	-3.696442000	2.565419000
H	2.032904000	-3.036232000	4.130001000
H	3.228872000	-1.244398000	2.913687000
H	2.312364000	-1.550342000	1.464302000
H	1.500850000	0.534103000	2.914159000

TS OAT S=1

Fe	-1.952783000	0.061914000	-0.146598000
N	-1.950876000	2.091930000	-0.162976000
N	-3.781127000	-0.002485000	0.677357000
N	-2.218120000	-1.869796000	-0.611849000
N	-3.049956000	0.376206000	-1.908243000
O	-1.063285000	-0.420275000	1.302523000
C	-2.436308000	1.594604000	-2.491938000
C	-2.202765000	2.603532000	-1.392284000
C	-2.217751000	3.979441000	-1.622610000
C	-1.963350000	4.852423000	-0.561442000
C	-1.721463000	4.317166000	0.705001000
C	-1.730407000	2.931768000	0.859034000
C	-4.480178000	0.609286000	-1.562676000
C	-4.819234000	0.169421000	-0.162160000
C	-6.138952000	0.002688000	0.262209000
C	-6.385821000	-0.342330000	1.591529000
C	-5.300441000	-0.523957000	2.453290000
C	-4.012604000	-0.355519000	1.954582000
C	-2.829783000	-0.848028000	-2.718889000
C	-2.741617000	-2.073198000	-1.839096000
C	-3.091216000	-3.352288000	-2.271415000
C	-2.874165000	-4.438257000	-1.417198000
C	-2.319177000	-4.211686000	-0.155103000
C	-2.009936000	-2.903805000	0.216098000
H	-3.052107000	2.018136000	-3.302934000
H	-1.461540000	1.307443000	-2.907447000
H	-2.433812000	4.359896000	-2.623044000
H	-1.971491000	5.933364000	-0.719753000
H	-5.142480000	0.121210000	-2.295342000
H	-4.687250000	1.688830000	-1.634050000
H	-6.959841000	0.142798000	-0.444281000
H	-7.409889000	-0.475931000	1.947515000
H	-1.856174000	-0.733726000	-3.216145000
H	-3.598480000	-0.965199000	-3.499938000
H	-3.522128000	-3.495940000	-3.264362000
H	-3.138860000	-5.449487000	-1.734826000
O	-0.398506000	0.108270000	-1.319248000
I	1.243094000	-0.806292000	-0.816977000
C	2.319689000	1.029402000	-0.464353000
C	3.640510000	1.019668000	-0.015594000
C	4.257057000	2.220852000	0.355373000
H	5.266082000	2.207550000	0.770783000
C	3.560735000	3.423459000	0.211425000
H	4.041590000	4.362862000	0.491532000
C	2.250936000	3.420346000	-0.279304000
H	1.707199000	4.360884000	-0.393341000
C	1.613042000	2.217771000	-0.607815000
H	0.579902000	2.191620000	-0.954564000
S	4.514699000	-0.540113000	0.233673000
O	3.447803000	-1.564676000	-0.099732000
O	5.100563000	-0.545519000	1.574639000
C	5.850332000	-0.743687000	-1.061960000
C	6.395853000	-2.153875000	-0.793364000

H	7.202334000	-2.347394000	-1.517404000
H	5.621047000	-2.921995000	-0.926293000
H	6.815884000	-2.236305000	0.219191000
C	6.911238000	0.330913000	-0.817412000
H	7.277917000	0.307550000	0.218794000
H	7.763714000	0.125946000	-1.483142000
H	6.543798000	1.339153000	-1.057805000
C	5.187438000	-0.622144000	-2.433500000
H	4.733883000	0.369659000	-2.587038000
H	5.964000000	-0.752098000	-3.202945000
H	4.425693000	-1.399540000	-2.587346000
H	-1.560635000	2.474949000	1.830604000
H	-1.537187000	4.957276000	1.569459000
H	-3.128129000	-0.522364000	2.563963000
H	-5.443915000	-0.804868000	3.497945000
H	-2.133430000	-5.032876000	0.539303000
H	-1.579960000	-2.635707000	1.183664000
C	-0.628737000	0.332637000	2.881701000
C	-0.687914000	-0.865472000	3.809211000
C	0.576277000	-1.727741000	3.740749000
C	1.818196000	-0.871858000	4.004000000
C	1.931600000	0.246850000	2.959081000
C	0.643343000	0.956863000	2.693083000
H	-1.480553000	1.013977000	2.988868000
H	-1.587908000	-1.462174000	3.594951000
H	-0.811256000	-0.477255000	4.837382000
H	0.507447000	-2.548354000	4.471267000
H	0.648977000	-2.190659000	2.740602000
H	2.733470000	-1.482325000	3.984420000
H	1.751423000	-0.431118000	5.013502000
H	2.719211000	0.971184000	3.223550000
H	2.284270000	-0.213011000	2.017686000
H	0.683779000	1.944674000	2.224555000

Epoxide S=2

Fe	1.719474000	0.220639000	-0.180027000
N	1.928594000	-1.678496000	-1.311187000
N	3.820041000	-0.218332000	0.347650000
N	2.127590000	2.356744000	0.257982000
N	2.826125000	0.900905000	-2.026007000
O	0.977428000	-0.558810000	1.699411000
C	2.306869000	-0.020498000	-3.052704000
C	2.323994000	-1.462850000	-2.581394000
C	2.682365000	-2.521496000	-3.420119000
C	2.618577000	-3.828845000	-2.929123000
C	2.210419000	-4.040626000	-1.609271000
C	1.880827000	-2.928913000	-0.834330000
C	4.288224000	0.814818000	-1.833005000
C	4.727432000	0.026670000	-0.610500000
C	6.061445000	-0.373937000	-0.470173000
C	6.455770000	-1.029245000	0.696100000
C	5.503951000	-1.273169000	1.692073000
C	4.195969000	-0.854301000	1.469983000
C	2.349091000	2.289762000	-2.171932000
C	2.447675000	3.044387000	-0.858170000
C	2.819341000	4.390148000	-0.802130000
C	2.850028000	5.037625000	0.436367000
C	2.522177000	4.316461000	1.586939000
C	2.172117000	2.973907000	1.446917000
H	2.847215000	0.077628000	-4.011258000
H	1.254427000	0.252657000	-3.225914000
H	3.007720000	-2.324154000	-4.443654000
H	2.894797000	-4.671411000	-3.567541000
H	4.691702000	1.834334000	-1.719288000
H	4.775990000	0.399627000	-2.731451000
H	6.780759000	-0.169226000	-1.266439000
H	7.492499000	-1.348322000	0.826646000
H	1.286267000	2.238653000	-2.451001000
H	2.885124000	2.834220000	-2.969534000
H	3.084098000	4.923410000	-1.717657000
H	3.136864000	6.089798000	0.502145000
O	-0.020953000	0.665776000	-1.082932000
I	-1.737200000	1.174248000	-0.332281000
C	-2.568408000	-0.809760000	-0.531687000
C	-3.863005000	-1.101095000	-0.104828000
C	-4.313075000	-2.427073000	-0.118209000
H	-5.304261000	-2.666510000	0.271452000
C	-3.475787000	-3.430904000	-0.613232000
H	-3.826689000	-4.464741000	-0.632798000
C	-2.192915000	-3.113780000	-1.073094000

H	-1.541730000	-3.899190000	-1.464022000
C	-1.719886000	-1.795962000	-1.023422000
H	-0.709482000	-1.532356000	-1.343987000
S	-4.926280000	0.197730000	0.564241000
O	-3.986674000	1.384976000	0.624341000
O	-5.539916000	-0.285481000	1.799809000
C	-6.245517000	0.637648000	-0.687803000
C	-6.973788000	1.822503000	-0.036409000
H	-7.783499000	2.136008000	-0.713320000
H	-6.301202000	2.678133000	0.117281000
H	-7.421397000	1.538619000	0.926685000
C	-7.162414000	-0.576260000	-0.846514000
H	-7.561607000	-0.908792000	0.122311000
H	-8.012584000	-0.281919000	-1.480972000
H	-6.656514000	-1.415933000	-1.345476000
C	-5.542798000	1.024999000	-1.988412000
H	-4.968421000	0.187102000	-2.413952000
H	-6.311768000	1.304170000	-2.725085000
H	-4.879210000	1.890464000	-1.850100000
H	1.574176000	-3.032402000	0.210174000
H	2.159925000	-5.044536000	-1.183751000
H	3.402062000	-1.032394000	2.197183000
H	5.768784000	-1.783242000	2.619812000
H	2.543711000	4.779928000	2.574828000
H	1.923363000	2.362950000	2.318642000
C	0.333492000	0.007604000	2.882596000
C	1.176464000	0.022947000	4.142550000
C	1.204279000	-1.332140000	4.872004000
C	1.336987000	-2.519304000	3.911515000
C	0.144266000	-2.580605000	2.948054000
C	-0.153284000	-1.255094000	2.295498000
H	-0.227325000	0.919928000	2.644145000
H	0.794961000	0.808817000	4.812889000
H	2.196981000	0.334074000	3.857043000
H	2.021436000	-1.335865000	5.608808000
H	0.270039000	-1.448106000	5.448138000
H	1.396218000	-3.461101000	4.477066000
H	2.277232000	-2.445875000	3.338542000
H	0.285650000	-3.349539000	2.169422000
H	-0.768824000	-2.870434000	3.497607000
H	-1.052516000	-1.219064000	1.671196000

Epoxide S=1

Fe	-1.830850000	-0.210855000	-0.262926000
N	-2.288354000	1.258826000	-1.533453000
N	-3.805716000	-0.177221000	0.340364000
N	-1.631892000	-2.014139000	0.616236000
N	-2.741543000	-1.458224000	-1.893098000
O	-1.117023000	1.104053000	1.506231000
C	-2.389450000	-0.638079000	-3.064150000
C	-2.594157000	0.838813000	-2.783160000
C	-3.030624000	1.736516000	-3.758349000
C	-3.143431000	3.093136000	-3.440337000
C	-2.829464000	3.512289000	-2.145566000
C	-2.411963000	2.557319000	-1.220488000
C	-4.185224000	-1.591160000	-1.644792000
C	-4.693214000	-0.833907000	-0.425125000
C	-6.056880000	-0.838827000	-0.111363000
C	-6.501961000	-0.149004000	1.015237000
C	-5.568007000	0.532043000	1.803706000
C	-4.229574000	0.491516000	1.429898000
C	-1.973212000	-2.699226000	-1.714884000
C	-1.808983000	-3.048092000	-0.244433000
C	-1.806875000	-4.369334000	0.206974000
C	-1.614708000	-4.632599000	1.566088000
C	-1.448151000	-3.559429000	2.444682000
C	-1.471002000	-2.266904000	1.926312000
H	-2.941658000	-0.937425000	-3.972540000
H	-1.315833000	-0.783782000	-3.253961000
H	-3.281853000	1.373632000	-4.757133000
H	-3.483514000	3.809912000	-4.191278000
H	-4.444513000	-2.655616000	-1.517606000
H	-4.753729000	-1.251656000	-2.526952000
H	-6.758938000	-1.380468000	-0.749595000
H	-7.562898000	-0.140776000	1.275469000
H	-0.970378000	-2.523448000	-2.130773000
H	-2.413708000	-3.554029000	-2.258241000
H	-1.960347000	-5.184715000	-0.502982000
H	-1.609734000	-5.660919000	1.934717000
O	-0.074166000	-0.319315000	-1.130708000

I	1.555816000	-0.919679000	-0.230254000
C	2.617768000	0.895103000	-0.722569000
C	3.926836000	1.104727000	-0.290754000
C	4.535102000	2.346484000	-0.512144000
H	5.536520000	2.535251000	-0.120829000
C	3.842250000	3.335129000	-1.216082000
H	4.317208000	4.301396000	-1.397846000
C	2.545176000	3.090593000	-1.679346000
H	2.009371000	3.863063000	-2.235902000
C	1.911526000	1.866897000	-1.423614000
H	0.886932000	1.664582000	-1.746014000
S	4.820281000	-0.168346000	0.628151000
O	3.752325000	-1.216324000	0.861640000
O	5.473452000	0.450461000	1.780284000
C	6.095483000	-0.961201000	-0.488815000
C	6.674378000	-2.090232000	0.376499000
H	7.450983000	-2.603958000	-0.210940000
H	5.905575000	-2.825952000	0.651745000
H	7.139643000	-1.696371000	1.291322000
C	7.149540000	0.095766000	-0.822539000
H	7.572170000	0.544101000	0.087917000
H	7.967801000	-0.397520000	-1.369442000
H	6.751168000	0.887954000	-1.473303000
C	5.369913000	-1.486325000	-1.727257000
H	4.900380000	-0.675805000	-2.306601000
H	6.110937000	-1.971432000	-2.381080000
H	4.609553000	-2.235938000	-1.465428000
H	-2.176864000	2.820342000	-0.188372000
H	-2.917348000	4.558892000	-1.848859000
H	-3.451991000	1.005986000	1.996972000
H	-5.870869000	1.086864000	2.693278000
H	-1.311398000	-3.715838000	3.516019000
H	-1.367297000	-1.394377000	2.571656000
C	-0.249766000	1.004354000	2.675358000
C	-0.937502000	1.176971000	4.016861000
C	-1.200194000	2.647914000	4.384725000
C	-1.671099000	3.480288000	3.187118000
C	-0.600801000	3.503285000	2.088343000
C	-0.107629000	2.123610000	1.727229000
H	0.471025000	0.181749000	2.589506000
H	-0.325285000	0.692785000	4.793664000
H	-1.886609000	0.612720000	3.980503000
H	-1.932199000	2.697048000	5.204775000
H	-0.268849000	3.091932000	4.776779000
H	-1.892695000	4.510687000	3.502751000
H	-2.613667000	3.070511000	2.783250000
H	-0.956164000	4.023185000	1.182816000
H	0.280455000	4.071860000	2.434673000
H	0.709207000	2.078030000	0.997519000

Epoxide_IO_bond_cleavage S=2

Fe	-1.888938000	0.098748000	0.019204000
N	-3.480449000	0.450053000	1.392256000
N	-3.049682000	1.074704000	-1.438018000
N	-0.283894000	0.660643000	-1.205041000
N	-1.584566000	2.218355000	0.648945000
O	-2.178796000	-1.771875000	-0.885991000
C	-1.922906000	2.172431000	2.086881000
C	-3.232137000	1.440612000	2.273531000
C	-4.141004000	1.743601000	3.287851000
C	-5.319640000	0.999756000	3.390454000
C	-5.567776000	-0.014646000	2.461617000
C	-4.618096000	-0.255861000	1.472504000
C	-2.504829000	3.081610000	-0.129868000
C	-3.088781000	2.419848000	-1.359513000
C	-3.707876000	3.183355000	-2.353477000
C	-4.306890000	2.540653000	-3.436891000
C	-4.264761000	1.144555000	-3.504317000
C	-3.617844000	0.451769000	-2.487205000
C	-0.153028000	2.480836000	0.389891000
C	0.300285000	1.832861000	-0.892733000
C	1.326837000	2.352264000	-1.681787000
C	1.760008000	1.626538000	-2.792183000
C	1.156522000	0.401919000	-3.089035000
C	0.126416000	-0.046372000	-2.268263000
H	-1.967221000	3.180660000	2.532654000
H	-1.126245000	1.607236000	2.594220000
H	-3.925285000	2.554514000	3.986585000
H	-6.041900000	1.217820000	4.180644000
H	-2.000455000	4.018887000	-0.415356000

H	-3.349322000	3.375175000	0.514545000
H	-3.720384000	4.272471000	-2.273018000
H	-4.797147000	3.121519000	-4.221652000
H	0.435481000	2.006865000	1.188921000
H	0.073012000	3.558836000	0.394972000
H	1.802541000	3.289570000	-1.394148000
H	2.572963000	2.006561000	-3.413416000
O	-0.949103000	-0.483666000	1.192981000
H	-4.761389000	-1.033220000	0.721351000
H	-6.482177000	-0.609197000	2.497030000
H	-3.534036000	-0.634640000	-2.503178000
H	-4.717144000	0.599914000	-4.334690000
H	1.475025000	-0.199008000	-3.941658000
H	-0.397364000	-0.984365000	-2.454208000
C	-2.801624000	-3.018705000	-1.372165000
C	-4.081386000	-3.439924000	-0.685640000
C	-3.869407000	-4.066977000	0.706593000
C	-2.727574000	-3.420288000	1.499974000
C	-1.397280000	-3.530330000	0.748618000
C	-1.500972000	-3.054947000	-0.675285000
H	-2.795651000	-3.053423000	-2.468267000
H	-4.602835000	-4.148276000	-1.348348000
H	-4.739232000	-2.553811000	-0.642387000
H	-4.811951000	-4.025524000	1.273508000
H	-3.640570000	-5.138320000	0.578115000
H	-2.632017000	-3.903022000	2.483578000
H	-2.936041000	-2.357057000	1.699335000
H	-0.605853000	-2.956366000	1.254658000
H	-1.061491000	-4.581680000	0.703850000
H	-0.588830000	-3.133607000	-1.278872000
I	2.332499000	-2.334360000	-0.006792000
C	3.614412000	-0.892560000	-0.925803000
C	3.879137000	0.403387000	-0.448339000
C	4.723713000	1.257579000	-1.178313000
H	4.894283000	2.269740000	-0.809426000
C	5.321790000	0.829293000	-2.362062000
H	5.984971000	1.500807000	-2.911491000
C	5.068049000	-0.461882000	-2.831415000
H	5.534142000	-0.817281000	-3.753471000
C	4.217020000	-1.312734000	-2.122184000
H	4.025959000	-2.318818000	-2.499686000
S	3.193208000	1.161870000	1.063400000
O	1.931577000	0.474381000	1.428529000
O	3.106454000	2.616816000	0.792079000
C	4.431161000	0.888250000	2.438190000
C	3.755197000	1.522792000	3.661631000
H	4.430441000	1.416078000	4.524490000
H	2.807307000	1.019802000	3.899847000
H	3.567318000	2.594491000	3.502095000
C	5.726896000	1.611423000	2.068910000
H	5.549315000	2.678539000	1.874043000
H	6.423324000	1.528713000	2.917646000
H	6.217494000	1.158757000	1.194751000
C	4.628193000	-0.617889000	2.607962000
H	5.077404000	-1.077133000	1.714268000
H	5.318887000	-0.785272000	3.448858000
H	3.681422000	-1.128046000	2.833045000

Epoxide_IO_bond_cleavage S=1

Fe	-2.029848000	-0.146083000	-0.182342000
N	-3.933173000	-0.319701000	-1.145854000
N	-3.114763000	-0.209306000	1.610056000
N	-0.438904000	-0.853082000	1.020840000
N	-2.452961000	-2.341093000	-0.082628000
O	-1.889204000	1.926822000	-0.068069000
C	-3.151302000	-2.602484000	-1.358849000
C	-4.219419000	-1.556992000	-1.594435000
C	-5.418206000	-1.817741000	-2.259338000
C	-6.319902000	-0.769991000	-2.468172000
C	-6.007592000	0.506571000	-1.991477000
C	-4.797476000	0.688927000	-1.326025000
C	-3.290419000	-2.618902000	1.112209000
C	-3.526886000	-1.424719000	2.017823000
C	-4.190838000	-1.585134000	3.237591000
C	-4.433182000	-0.467416000	4.036429000
C	-4.003961000	0.788986000	3.596572000
C	-3.344040000	0.874558000	2.375666000
C	-1.099495000	-2.935099000	-0.014949000
C	-0.230981000	-2.180502000	0.959766000
C	0.786896000	-2.780113000	1.700121000

C	1.602395000	-1.980160000	2.502500000
C	1.373992000	-0.602979000	2.550932000
C	0.339335000	-0.075226000	1.786207000
H	-3.578014000	-3.619145000	-1.394107000
H	-2.404798000	-2.519052000	-2.163463000
H	-5.639615000	-2.827948000	-2.609916000
H	-7.262017000	-0.951017000	-2.990930000
H	-2.839127000	-3.432274000	1.703084000
H	-4.274183000	-2.993555000	0.785902000
H	-4.515254000	-2.579052000	3.553894000
H	-4.951139000	-0.575001000	4.992361000
H	-0.618669000	-2.816164000	-0.998067000
H	-1.138998000	-4.009487000	0.230006000
H	0.946568000	-3.857342000	1.629863000
H	2.417700000	-2.426076000	3.076112000
O	-1.241524000	-0.270379000	-1.585356000
H	-4.503999000	1.658460000	-0.917443000
H	-6.690677000	1.347133000	-2.125380000
H	-2.979536000	1.823013000	1.976013000
H	-4.174111000	1.689095000	4.189619000
H	1.996826000	0.060263000	3.151812000
H	0.129650000	0.994615000	1.774919000
C	-0.596856000	2.586433000	-0.467868000
C	-0.131550000	3.662973000	0.487065000
C	-0.835575000	5.013861000	0.278891000
C	-2.339522000	4.848934000	0.035509000
C	-2.597540000	4.035919000	-1.240383000
C	-1.800528000	2.759417000	-1.290651000
H	0.134158000	1.835415000	-0.786001000
H	0.959841000	3.761425000	0.375338000
H	-0.296263000	3.295968000	1.515476000
H	-0.650487000	5.664441000	1.146494000
H	-0.388127000	5.524956000	-0.591188000
H	-2.824975000	5.831822000	-0.054969000
H	-2.809531000	4.346279000	0.899286000
H	-3.669439000	3.813584000	-1.374641000
H	-2.297492000	4.618949000	-2.129517000
H	-1.879314000	2.146344000	-2.195865000
I	3.952700000	1.774617000	0.851745000
C	4.294857000	-0.326960000	0.655482000
C	3.615374000	-1.218548000	-0.197085000
C	3.939952000	-2.586095000	-0.185154000
H	3.385656000	-3.263455000	-0.835449000
C	4.942070000	-3.073941000	0.650988000
H	5.190218000	-4.137326000	0.642294000
C	5.623475000	-2.191061000	1.492096000
H	6.415378000	-2.553906000	2.151537000
C	5.299414000	-0.832307000	1.496509000
H	5.838427000	-0.155338000	2.161754000
S	2.262706000	-0.790969000	-1.337120000
O	1.592234000	0.430658000	-0.834886000
O	1.448465000	-2.021477000	-1.486039000
C	3.001968000	-0.404551000	-3.009625000
C	1.782409000	-0.040394000	-3.867913000
H	2.131929000	0.186258000	-4.886917000
H	1.264858000	0.845266000	-3.473143000
H	1.068211000	-0.874208000	-3.921951000
C	3.700129000	-1.665873000	-3.520379000
H	3.006173000	-2.516555000	-3.570616000
H	4.076454000	-1.467409000	-4.535804000
H	4.563292000	-1.939395000	-2.895683000
C	3.965168000	0.770970000	-2.853021000
H	4.817833000	0.520278000	-2.204317000
H	4.367275000	1.025975000	-3.845713000
H	3.460832000	1.658224000	-2.446650000

Intramolecular HAT S=2

O	1.825626000	1.988814000	0.043244000
C	0.619846000	2.678533000	0.471617000
C	0.270686000	3.908637000	-0.351124000
C	0.918134000	5.196974000	0.180643000
C	2.405184000	5.007518000	0.538205000
C	2.579749000	3.899152000	1.524276000
C	1.747338000	2.723500000	1.426836000
H	-0.199224000	1.984959000	0.699243000
H	-0.824311000	4.018665000	-0.380640000
H	0.807190000	6.002733000	-0.560023000
H	0.380457000	5.525333000	1.085767000
H	2.833911000	5.941681000	0.931200000
H	2.976663000	4.778985000	-0.386521000

H	3.321840000	3.957633000	2.323837000
H	1.756318000	1.975292000	2.223660000
H	0.592350000	3.717347000	-1.389640000
Fe	2.020753000	-0.190214000	0.127238000
N	3.769853000	-0.423938000	1.195033000
N	3.211306000	-0.242666000	-1.553340000
N	0.483480000	-0.697668000	-1.121296000
N	2.347441000	-2.429037000	-0.053075000
C	2.961793000	-2.720100000	1.254804000
C	4.013493000	-1.686024000	1.604166000
C	5.156301000	-1.985472000	2.347202000
C	6.041853000	-0.956354000	2.679341000
C	5.771230000	0.345261000	2.247179000
C	4.620210000	0.568805000	1.496034000
C	3.222995000	-2.690023000	-1.211787000
C	3.625898000	-1.447235000	-1.989571000
C	4.423328000	-1.556385000	-3.133358000
C	4.793009000	-0.403012000	-3.823929000
C	4.355009000	0.840361000	-3.354780000
C	3.562635000	0.878432000	-2.213697000
C	0.960565000	-2.903394000	-0.210776000
C	0.177315000	-2.010677000	-1.147323000
C	-0.858222000	-2.482223000	-1.953354000
C	-1.581157000	-1.577947000	-2.732872000
C	-1.242274000	-0.222796000	-2.700115000
C	-0.196895000	0.178470000	-1.876560000
H	3.386620000	-3.737642000	1.301802000
H	2.166908000	-2.644726000	2.011854000
H	5.346430000	-3.013049000	2.663845000
H	6.939297000	-1.170550000	3.264473000
H	2.731220000	-3.391721000	-1.905785000
H	4.143189000	-3.197266000	-0.877287000
H	4.749639000	-2.541666000	-3.473953000
H	5.416233000	-0.471698000	-4.718630000
H	0.454181000	-2.843554000	0.764050000
H	0.913086000	-3.953420000	-0.546131000
H	-1.101983000	-3.545623000	-1.951206000
H	-2.407437000	-1.926638000	-3.355958000
O	1.078987000	-0.304597000	1.609111000
H	4.358980000	1.561228000	1.119975000
H	6.442374000	1.173636000	2.480123000
H	3.184456000	1.812656000	-1.793300000
H	4.621799000	1.767722000	-3.864310000
H	-1.784233000	0.516753000	-3.290422000
H	0.111546000	1.222245000	-1.807393000
H	0.102814000	-0.240964000	1.463488000
I	-3.479923000	2.037116000	-0.484548000
C	-4.146031000	0.033711000	-0.808979000
C	-3.709746000	-1.122457000	-0.132753000
C	-4.224324000	-2.382521000	-0.484891000
H	-3.856573000	-3.267979000	0.034943000
C	-5.179549000	-2.503682000	-1.492012000
H	-5.577597000	-3.487240000	-1.749823000
C	-5.621028000	-1.359127000	-2.160004000
H	-6.373049000	-1.434062000	-2.948965000
C	-5.105690000	-0.104655000	-1.824133000
H	-5.458652000	0.779476000	-2.358079000
S	-2.471126000	-1.193112000	1.190983000
O	-1.544255000	-0.031416000	1.037898000
O	-1.851746000	-2.537170000	1.132975000
C	-3.342242000	-1.029334000	2.838074000
C	-2.197373000	-1.082425000	3.859141000
H	-2.632226000	-1.000194000	4.867028000
H	-1.492823000	-0.249696000	3.720691000
H	-1.649011000	-2.033221000	3.797975000
C	-4.289837000	-2.221662000	2.980265000
H	-3.752039000	-3.176483000	2.896263000
H	-4.753779000	-2.175617000	3.977548000
H	-5.100398000	-2.194344000	2.237096000
C	-4.077883000	0.309022000	2.866466000
H	-4.871110000	0.359750000	2.105766000
H	-4.556650000	0.418000000	3.851827000
H	-3.391431000	1.154361000	2.722256000

Hydroxyepoxide S=2

Fe	-2.205746000	-0.005674000	-0.212277000
N	-3.994410000	-0.775405000	0.776040000
N	-3.246792000	1.740988000	-0.931497000
N	-0.434578000	1.270641000	-0.373867000
N	-2.307001000	1.183657000	1.688958000

O	-1.683222000	-1.004619000	-2.084738000
C	-2.835257000	0.209731000	2.665310000
C	-4.043729000	-0.503648000	2.096330000
C	-5.143158000	-0.872272000	2.873675000
C	-6.212194000	-1.536464000	2.265790000
C	-6.159709000	-1.798388000	0.893752000
C	-5.029297000	-1.395889000	0.186844000
C	-3.232858000	2.316823000	1.454613000
C	-3.413257000	2.702239000	-0.004317000
C	-3.820648000	3.990839000	-0.361260000
C	-4.069934000	4.277412000	-1.705263000
C	-3.898254000	3.270376000	-2.659552000
C	-3.477057000	2.015989000	-2.225552000
C	-0.923845000	1.601059000	1.983016000
C	-0.149661000	1.972104000	0.738649000
C	0.868024000	2.926881000	0.753423000
C	1.603335000	3.151736000	-0.411857000
C	1.294773000	2.423414000	-1.562822000
C	0.261623000	1.492300000	-1.498119000
H	-3.079347000	0.674549000	3.637053000
H	-2.046271000	-0.538265000	2.843386000
H	-5.163005000	-0.636273000	3.939614000
H	-7.082708000	-1.834554000	2.854912000
H	-2.927997000	3.199952000	2.040787000
H	-4.228865000	2.031564000	1.830551000
H	-3.944008000	4.759326000	0.404984000
H	-4.391239000	5.277750000	-2.004891000
H	-0.387634000	0.757349000	2.443221000
H	-0.891464000	2.424125000	2.717910000
H	1.088595000	3.472414000	1.672212000
H	2.414171000	3.883192000	-0.418941000
H	-4.940664000	-1.571645000	-0.888780000
H	-6.980207000	-2.300189000	0.378070000
H	-3.303584000	1.196414000	-2.928369000
H	-4.080415000	3.453804000	-3.719825000
H	1.845659000	2.566315000	-2.493410000
H	-0.032486000	0.899814000	-2.367222000
C	-1.523490000	-2.162458000	-2.971848000
C	-2.576535000	-3.249035000	-2.906892000
C	-2.289830000	-4.263170000	-1.790464000
C	-2.021295000	-3.555614000	-0.461666000
C	-0.778512000	-2.664063000	-0.519654000
C	-0.598452000	-1.944920000	-1.847340000
H	-1.190733000	-1.844041000	-3.966281000
H	-2.625054000	-3.749598000	-3.885611000
H	-3.559655000	-2.769132000	-2.758188000
H	-3.136491000	-4.957878000	-1.687096000
H	-1.417781000	-4.879193000	-2.071238000
H	-1.909366000	-4.271751000	0.365308000
H	-2.880474000	-2.918651000	-0.204947000
H	0.132117000	-3.253277000	-0.322465000
H	0.396336000	-1.516416000	-2.020562000
O	-0.902683000	-1.640677000	0.488475000
H	0.015755000	-1.308786000	0.678566000
I	3.535121000	-0.802910000	-1.985049000
C	4.128785000	0.649072000	-0.535275000
C	3.692966000	0.743555000	0.801029000
C	4.164550000	1.779037000	1.626937000
H	3.796870000	1.848440000	2.651372000
C	5.076753000	2.713948000	1.142498000
H	5.441362000	3.508560000	1.796617000
C	5.517706000	2.620494000	-0.179448000
H	6.236492000	3.341666000	-0.575525000
C	5.044749000	1.601099000	-1.009558000
H	5.397204000	1.544594000	-2.041023000
S	2.508755000	-0.349159000	1.629932000
O	1.592150000	-0.922460000	0.595630000
O	1.867844000	0.433137000	2.709413000
C	3.443008000	-1.762465000	2.424375000
C	2.338325000	-2.603604000	3.078585000
H	2.810084000	-3.463977000	3.577374000
H	1.628124000	-2.989466000	2.332565000
C	4.389734000	-1.159420000	3.463898000
H	3.844981000	-0.559244000	4.206186000
H	4.891874000	-1.984749000	3.991751000
H	5.171059000	-0.540566000	2.998563000
C	4.186128000	-2.532763000	1.335155000
H	4.949598000	-1.914158000	0.840725000
H	4.703063000	-3.382983000	1.806259000
H	3.500379000	-2.926494000	0.572703000

H	1.789471000	-2.026085000	3.835962000

Hydroxyepoxide S=1

Fe	-2.158689000	0.119513000	-0.101312000
N	-3.815395000	-0.679847000	0.662497000
N	-3.281413000	1.654302000	-0.836260000
N	-0.546569000	1.228235000	-0.441292000
N	-2.244365000	1.292719000	1.725932000
O	-1.842269000	-1.183131000	-1.961116000
C	-2.786692000	0.272786000	2.649364000
C	-3.947427000	-0.448282000	1.990654000
C	-5.085996000	-0.857322000	2.684944000
C	-6.108439000	-1.514147000	1.993691000
C	-5.971023000	-1.725052000	0.619228000
C	-4.807361000	-1.287578000	-0.008132000
C	-3.158075000	2.431235000	1.491571000
C	-3.543722000	2.643418000	0.033882000
C	-4.198655000	3.808212000	-0.376654000
C	-4.588108000	3.936074000	-1.710645000
C	-4.309996000	2.896169000	-2.604398000
C	-3.648706000	1.770189000	-2.124560000
C	-0.836532000	1.674422000	1.938527000
C	-0.133707000	1.951035000	0.625473000
C	0.934333000	2.840503000	0.521587000
C	1.589609000	2.980109000	-0.703463000
C	1.147407000	2.236148000	-1.799039000
C	0.069414000	1.373350000	-1.625073000
H	-3.090114000	0.696113000	3.622625000
H	-1.986843000	-0.460400000	2.835732000
H	-5.172548000	-0.656106000	3.754624000
H	-7.007417000	-1.843211000	2.519887000
H	-2.723659000	3.361959000	1.891954000
H	-4.088981000	2.265456000	2.057917000
H	-4.401202000	4.603545000	0.344274000
H	-5.101233000	4.838398000	-2.051117000
H	-0.306491000	0.839842000	2.420990000
H	-0.731978000	2.534691000	2.621311000
H	1.257742000	3.399717000	1.400282000
H	2.440248000	3.658163000	-0.798982000
H	-4.645234000	-1.421299000	-1.079484000
H	-6.753108000	-2.216564000	0.038089000
H	-3.390102000	0.929040000	-2.773370000
H	-4.596361000	2.957296000	-3.655650000
H	1.629749000	2.314531000	-2.774175000
H	-0.328118000	0.771184000	-2.444306000
C	-1.632763000	-2.437902000	-2.687654000
C	-2.587015000	-3.586158000	-2.428415000
C	-2.178943000	-4.411307000	-1.199302000
C	-1.920922000	-3.511964000	0.011366000
C	-0.758603000	-2.549608000	-0.233900000
C	-0.688591000	-1.998285000	-1.648052000
H	-1.356747000	-2.243804000	-3.730533000
H	-2.620641000	-4.221511000	-3.326286000
H	-3.604070000	-3.177357000	-2.305028000
H	-2.961124000	-5.148167000	-0.963969000
H	-1.269651000	-4.991389000	-1.435567000
H	-1.710356000	-4.102966000	0.914773000
H	-2.818603000	-2.918272000	0.226570000
H	0.202350000	-3.042220000	-0.013686000
H	0.270213000	-1.541786000	-1.923841000
O	-0.884035000	-1.408390000	0.648874000
H	0.043328000	-1.067243000	0.774503000
I	3.462782000	-0.887696000	-2.003742000
C	4.165193000	0.631673000	-0.676943000
C	3.788309000	0.828148000	0.666631000
C	4.326847000	1.897520000	1.403798000
H	4.004858000	2.045905000	2.435287000
C	5.247254000	2.767268000	0.822863000
H	5.663346000	3.589905000	1.407935000
C	5.629868000	2.573027000	-0.506305000
H	6.353656000	3.242305000	-0.977244000
C	5.091110000	1.518591000	-1.248069000
H	5.398682000	1.383571000	-2.286584000
S	2.600961000	-0.163546000	1.609428000
O	1.622797000	-0.763630000	0.647784000
O	2.029917000	0.707347000	2.659771000
C	3.512731000	-1.561128000	2.455229000
C	2.404051000	-2.321517000	3.195714000
H	2.864307000	-3.165571000	3.731694000
H	1.656741000	-2.727790000	2.498325000

C	4.516155000	-0.932067000	3.423842000
H	4.019147000	-0.270892000	4.147555000
H	5.009932000	-1.742413000	3.981926000
H	5.299819000	-0.369153000	2.895628000
C	4.189500000	-2.420603000	1.389767000
H	4.959299000	-1.861748000	0.837438000
H	4.688761000	-3.263056000	1.892769000
H	3.464552000	-2.828295000	0.672185000
H	1.900804000	-1.680310000	3.933338000

Dehydration S=2

Fe	-2.200434000	-0.101108000	-0.164265000
N	-4.112507000	-0.825858000	0.650539000
N	-3.153532000	1.713637000	-0.909258000
N	-0.373938000	1.158910000	-0.215924000
N	-2.428218000	1.081225000	1.733778000
O	-2.228950000	-1.080011000	-2.067639000
C	-3.030279000	0.081954000	2.639623000
C	-4.212757000	-0.602778000	1.977014000
C	-5.342854000	-0.994964000	2.698495000
C	-6.388811000	-1.635257000	2.028220000
C	-6.284659000	-1.845735000	0.650800000
C	-5.126843000	-1.417351000	0.003624000
C	-3.324031000	2.230087000	1.475200000
C	-3.393576000	2.654971000	0.019797000
C	-3.776553000	3.949957000	-0.341789000
C	-3.928242000	4.259623000	-1.694800000
C	-3.687393000	3.268825000	-2.651995000
C	-3.293100000	2.008418000	-2.211552000
C	-1.057578000	1.482597000	2.100814000
C	-0.238042000	1.906952000	0.897226000
C	0.656801000	2.976949000	0.950442000
C	1.430537000	3.273225000	-0.174734000
C	1.290740000	2.486753000	-1.319089000
C	0.372903000	1.438534000	-1.291536000
H	-3.333428000	0.519977000	3.607295000
H	-2.264399000	-0.681818000	2.844684000
H	-5.404670000	-0.796716000	3.770628000
H	-7.281017000	-1.952455000	2.573203000
H	-3.052610000	3.091902000	2.108280000
H	-4.346620000	1.944577000	1.770378000
H	-3.959199000	4.703067000	0.427853000
H	-4.229851000	5.264510000	-1.999648000
H	-0.549033000	0.608705000	2.536065000
H	-1.056110000	2.278120000	2.866540000
H	0.746375000	3.570758000	1.861775000
H	2.134680000	4.107328000	-0.155622000
H	-5.003791000	-1.540041000	-1.075568000
H	-7.086404000	-2.324814000	0.086089000
H	-3.073639000	1.193888000	-2.906394000
H	-3.796685000	3.470377000	-3.718972000
H	1.881389000	2.674541000	-2.216416000
H	0.224859000	0.799304000	-2.164119000
C	-2.677589000	-2.289377000	-2.743131000
C	-2.691298000	-3.553805000	-1.919516000
C	-1.344220000	-3.954309000	-1.378304000
C	-0.191481000	-3.366013000	-1.731141000
C	-0.082993000	-2.230149000	-2.709162000
C	-1.407431000	-1.646933000	-3.128883000
H	-3.560247000	-2.111370000	-3.368970000
H	-3.088058000	-4.365824000	-2.556032000
H	-3.421906000	-3.447228000	-1.098306000
H	-1.330705000	-4.804017000	-0.688545000
H	0.753065000	-3.726554000	-1.317308000
H	0.564870000	-1.444785000	-2.286851000
H	0.435593000	-2.582145000	-3.619630000
H	-1.397302000	-1.016762000	-4.026311000
O	-1.169948000	-1.678904000	0.757908000
H	-0.964570000	-2.435022000	0.170264000
H	-0.322997000	-1.463517000	1.232513000
I	4.354772000	0.241699000	-1.738550000
C	4.016555000	1.003052000	0.223240000
C	3.178773000	0.445987000	1.209836000
C	3.031063000	1.079453000	2.456711000
H	2.370300000	0.636715000	3.202240000
C	3.720210000	2.256217000	2.739711000
H	3.607597000	2.732834000	3.715668000
C	4.559275000	2.809587000	1.769568000
H	5.114862000	3.727171000	1.977159000
C	4.699340000	2.193015000	0.524425000

H	5.355113000	2.640497000	-0.224692000
S	2.252466000	-1.101420000	1.046588000
O	1.897340000	-1.373818000	-0.360272000
O	1.085962000	-0.983927000	1.988754000
C	3.326250000	-2.491624000	1.700922000
C	2.454749000	-3.741680000	1.523381000
H	3.007229000	-4.605577000	1.923091000
H	2.243584000	-3.936189000	0.462804000
C	3.621389000	-2.209426000	3.174747000
H	2.696826000	-2.106598000	3.759909000
H	4.190406000	-3.060879000	3.578762000
H	4.238572000	-1.308979000	3.306753000
C	4.595197000	-2.553401000	0.852022000
H	5.205863000	-1.644501000	0.957226000
H	5.201896000	-3.402751000	1.202087000
H	4.368596000	-2.708791000	-0.211661000
H	1.508292000	-3.657682000	2.077379000

Dehydration S=1

Fe	-2.306568000	0.030373000	-0.072372000
N	-4.028163000	-0.529942000	0.771438000
N	-3.237117000	1.699119000	-0.840803000
N	-0.584478000	0.942909000	-0.487693000
N	-2.218296000	1.302159000	1.724256000
O	-2.487344000	-1.246631000	-1.886531000
C	-2.863566000	0.405396000	2.704330000
C	-4.098217000	-0.231527000	2.091957000
C	-5.251425000	-0.497605000	2.831239000
C	-6.354480000	-1.076592000	2.197621000
C	-6.282496000	-1.347255000	0.829088000
C	-5.100963000	-1.049425000	0.154246000
C	-2.979771000	2.537170000	1.454038000
C	-3.347126000	2.747155000	-0.008057000
C	-3.840211000	3.976541000	-0.455783000
C	-4.231498000	4.109100000	-1.788471000
C	-4.116943000	3.007464000	-2.643200000
C	-3.609089000	1.819564000	-2.127295000
C	-0.766669000	1.503000000	1.877201000
C	-0.098489000	1.693301000	0.530495000
C	0.978800000	2.556452000	0.340201000
C	1.574298000	2.640895000	-0.920965000
C	1.072720000	1.856741000	-1.958855000
C	-0.011797000	1.024429000	-1.697731000
H	-3.122334000	0.919955000	3.646236000
H	-2.148886000	-0.395731000	2.941028000
H	-5.286444000	-0.243884000	3.892712000
H	-7.264664000	-1.293606000	2.761324000
H	-2.431410000	3.420300000	1.822650000
H	-3.921146000	2.507379000	2.026547000
H	-3.917986000	4.817905000	0.236618000
H	-4.619982000	5.061046000	-2.157622000
H	-0.338109000	0.593750000	2.324929000
H	-0.527548000	2.345275000	2.549685000
H	1.353453000	3.149340000	1.176303000
H	2.423445000	3.306143000	-1.087713000
H	-4.999424000	-1.212744000	-0.919662000
H	-7.129216000	-1.772441000	0.287459000
H	-3.480793000	0.924801000	-2.741192000
H	-4.411459000	3.068292000	-3.692251000
H	1.515432000	1.881503000	-2.955065000
H	-0.452452000	0.405203000	-2.479157000
C	-3.027145000	-2.439070000	-2.523728000
C	-3.317875000	-3.628376000	-1.640459000
C	-2.124638000	-4.141842000	-0.881635000
C	-0.864324000	-3.749221000	-1.120730000
C	-0.472328000	-2.729059000	-2.154314000
C	-1.641170000	-2.014157000	-2.785836000
H	-3.793124000	-2.201419000	-3.272021000
H	-3.706285000	-4.433816000	-2.291426000
H	-4.147213000	-3.395873000	-0.951554000
H	-2.317593000	-4.918110000	-0.134688000
H	-0.045686000	-4.209225000	-0.560584000
H	0.230881000	-1.997450000	-1.721176000
H	0.095006000	-3.228910000	-2.960719000
H	-1.432432000	-1.485945000	-3.724332000
O	-1.349440000	-1.515853000	0.897776000
H	-1.508629000	-2.409850000	0.538362000
H	-0.377134000	-1.456296000	1.096492000
I	4.739184000	0.606305000	-1.556620000
C	4.205153000	1.041129000	0.460196000

C	3.292416000	0.329253000	1.264670000
C	3.026103000	0.750829000	2.579504000
H	2.315810000	0.186723000	3.184120000
C	3.657405000	1.875610000	3.105533000
H	3.449857000	2.188152000	4.131025000
C	4.562070000	2.587545000	2.313517000
H	5.071367000	3.468635000	2.710899000
C	4.830189000	2.174409000	1.006899000
H	5.541284000	2.739900000	0.401854000
S	2.370200000	-1.146573000	0.751374000
O	2.101029000	-1.126056000	-0.699323000
O	1.158109000	-1.188651000	1.639411000
C	3.393660000	-2.654793000	1.194733000
C	2.530290000	-3.834460000	0.731852000
H	3.065458000	-4.766887000	0.967722000
H	2.361418000	-3.803100000	-0.353919000
C	3.596701000	-2.655062000	2.710619000
H	2.638135000	-2.653398000	3.248012000
H	4.142538000	-3.571529000	2.983086000
H	4.201843000	-1.799693000	3.044720000
C	4.714712000	-2.584657000	0.430923000
H	5.324390000	-1.723329000	0.740572000
H	5.289945000	-3.495765000	0.657037000
H	4.557769000	-2.538437000	-0.655195000
H	1.564483000	-3.855024000	1.257546000

H2O loss S=2

Fe	1.448362000	0.020531000	-0.296041000
N	0.447946000	-1.035061000	-1.907378000
N	3.204485000	0.707313000	-1.358794000
N	1.644521000	1.766285000	0.959777000
N	0.547835000	1.685160000	-1.615702000
O	2.637240000	-1.500277000	0.676835000
C	-0.694084000	1.107599000	-2.158069000
C	-0.497327000	-0.336907000	-2.568096000
C	-1.273758000	-0.933349000	-3.565360000
C	-1.060146000	-2.275279000	-3.883666000
C	-0.062917000	-2.984773000	-3.207469000
C	0.666936000	-2.321603000	-2.226000000
C	1.553511000	1.911639000	-2.678098000
C	2.985440000	1.712681000	-2.227574000
C	4.038127000	2.462270000	-2.755993000
C	5.348224000	2.143701000	-2.386313000
C	5.566512000	1.086857000	-1.499071000
C	4.461674000	0.397152000	-1.002098000
C	0.316631000	2.876133000	-0.775784000
C	1.180947000	2.928353000	0.466229000
C	1.409794000	4.138476000	1.129829000
C	2.115914000	4.135848000	2.332523000
C	2.585836000	2.920389000	2.841591000
C	2.331416000	1.762523000	2.115088000
H	-1.091509000	1.689514000	-3.009641000
H	-1.455705000	1.143068000	-1.366056000
H	-2.030529000	-0.345866000	-4.089149000
H	-1.654908000	-2.758681000	-4.662209000
H	1.427451000	2.906662000	-3.138333000
H	1.371932000	1.174483000	-3.476969000
H	3.836323000	3.278911000	-3.452352000
H	6.188321000	2.714994000	-2.788335000
H	-0.722967000	2.849830000	-0.419778000
H	0.437374000	3.806672000	-1.356513000
H	1.030364000	5.071204000	0.707005000
H	2.301800000	5.070989000	2.866000000
H	1.463623000	-2.826275000	-1.674866000
H	0.151414000	-4.029031000	-3.440954000
H	4.566685000	-0.432118000	-0.297795000
H	6.574120000	0.802444000	-1.190990000
H	3.145587000	2.872351000	3.777134000
H	2.692161000	0.790657000	2.457630000
C	2.650334000	-1.941011000	2.070179000
C	4.006088000	-1.939371000	2.728771000
C	5.034449000	-2.760543000	1.995808000
C	4.743094000	-3.624395000	1.016264000
C	3.347894000	-3.892980000	0.515494000
C	2.330961000	-2.889418000	0.996297000
H	1.810606000	-1.526179000	2.638359000
H	3.878392000	-2.322186000	3.758730000
H	4.354671000	-0.897515000	2.849685000
H	6.071927000	-2.646140000	2.322472000
H	5.545877000	-4.204757000	0.552814000

H	3.333652000	-3.929941000	-0.589182000
H	3.014314000	-4.895148000	0.844025000
H	1.273275000	-3.127589000	0.834783000
I	-4.546279000	1.302229000	-0.164159000
C	-3.735508000	-0.658667000	-0.361666000
C	-2.539586000	-1.142584000	0.210306000
C	-2.115218000	-2.460595000	-0.038095000
H	-1.179967000	-2.807928000	0.398591000
C	-2.874585000	-3.311616000	-0.835205000
H	-2.538653000	-4.334595000	-1.015068000
C	-4.066591000	-2.844410000	-1.394873000
H	-4.680253000	-3.500787000	-2.016305000
C	-4.486207000	-1.533187000	-1.165679000
H	-5.416334000	-1.181886000	-1.616108000
S	-1.445102000	-0.195475000	1.298079000
O	-1.552114000	1.248116000	1.022107000
O	-0.070850000	-0.793858000	1.103217000
C	-1.912351000	-0.517552000	3.079566000
C	-0.927817000	0.349746000	3.875571000
H	-1.157265000	0.232724000	4.945674000
H	-1.025570000	1.412928000	3.615808000
H	0.113250000	0.035659000	3.712134000
C	-1.731877000	-2.008713000	3.363594000
H	-0.700379000	-2.337821000	3.173532000
H	-1.955853000	-2.183958000	4.427078000
H	-2.424168000	-2.628132000	2.775047000
C	-3.359495000	-0.058307000	3.262146000
H	-4.059534000	-0.651998000	2.655549000
H	-3.633021000	-0.199786000	4.319006000
H	-3.485087000	1.004516000	3.014338000

H2O loss S=1

Fe	-1.437074000	0.163420000	0.296105000
N	-0.666306000	-0.926514000	1.779965000
N	-3.229930000	0.648786000	1.321909000
N	-1.902799000	1.655897000	-0.943476000
N	-0.648553000	1.743994000	1.493829000
O	-2.493339000	-1.434251000	-0.745365000
C	0.576748000	1.147361000	2.073869000
C	0.293722000	-0.281146000	2.487232000
C	0.970622000	-0.907217000	3.533582000
C	0.647993000	-2.224948000	3.864905000
C	-0.365738000	-2.870622000	3.153081000
C	-0.999153000	-2.181496000	2.122388000
C	-1.659354000	2.046291000	2.541247000
C	-3.082149000	1.689624000	2.158164000
C	-4.182963000	2.339662000	2.719328000
C	-5.466180000	1.877459000	2.414771000
C	-5.607987000	0.784262000	1.555829000
C	-4.459700000	0.202435000	1.020777000
C	-0.407451000	2.868631000	0.561159000
C	-1.387139000	2.857017000	-0.589679000
C	-1.693831000	4.018386000	-1.302044000
C	-2.543726000	3.941979000	-2.406004000
C	-3.080092000	2.700218000	-2.757318000
C	-2.739450000	1.588933000	-1.993305000
H	0.963692000	1.730169000	2.927673000
H	1.359493000	1.161692000	1.303024000
H	1.736145000	-0.360339000	4.087446000
H	1.166500000	-2.732475000	4.681400000
H	-1.592156000	3.102429000	2.848983000
H	-1.411821000	1.445746000	3.430979000
H	-4.037453000	3.189918000	3.389239000
H	-6.344273000	2.366752000	2.842555000
H	0.595095000	2.740040000	0.128822000
H	-0.426780000	3.840882000	1.080870000
H	-1.266323000	4.973393000	-0.989834000
H	-2.792740000	4.839564000	-2.976571000
H	-1.812100000	-2.629748000	1.553091000
H	-0.672800000	-3.889109000	3.396982000
H	-4.504255000	-0.641713000	0.328150000
H	-6.592092000	0.390626000	1.295586000
H	-3.762106000	2.592764000	-3.602440000
H	-3.158125000	0.608014000	-2.205773000
C	-2.440652000	-1.850905000	-2.142336000
C	-3.767152000	-1.986774000	-2.849792000
C	-4.743181000	-2.889795000	-2.143591000
C	-4.399466000	-3.737958000	-1.167898000
C	-2.993164000	-3.896852000	-0.653903000
C	-2.063133000	-2.784170000	-1.073184000

H	-1.631797000	-1.350718000	-2.685223000
H	-3.558588000	-2.374944000	-3.864733000
H	-4.213142000	-0.990132000	-3.015004000
H	-5.780510000	-2.853961000	-2.488172000
H	-5.158930000	-4.386903000	-0.722849000
H	-2.994068000	-3.992609000	0.446428000
H	-2.561248000	-4.846289000	-1.022928000
H	-0.993922000	-2.932841000	-0.888764000
I	4.777615000	1.252198000	0.194340000
C	3.810496000	-0.621854000	0.488707000
C	2.590839000	-1.044680000	-0.081788000
C	2.062426000	-2.311676000	0.226237000
H	1.109715000	-2.608733000	-0.207835000
C	2.739413000	-3.173905000	1.082519000
H	2.322639000	-4.157246000	1.307887000
C	3.952504000	-2.767378000	1.644950000
H	4.502199000	-3.432882000	2.314684000
C	4.475234000	-1.506084000	1.356433000
H	5.420924000	-1.201911000	1.808780000
S	1.583230000	-0.075081000	-1.233667000
O	1.803220000	1.369443000	-1.035884000
O	0.166298000	-0.545840000	-1.020323000
C	2.036362000	-0.532298000	-2.989700000
C	1.133142000	0.365863000	-3.845218000
H	1.355048000	0.163557000	-4.904171000
H	1.323891000	1.430357000	-3.650839000
H	0.067790000	0.155610000	-3.671026000
C	1.739303000	-2.018019000	-3.192673000
H	0.683420000	-2.253249000	-2.998129000
H	1.956951000	-2.269895000	-4.241968000
H	2.375043000	-2.656367000	-2.562155000
C	3.518036000	-0.202410000	-3.175879000
H	4.161471000	-0.816337000	-2.528128000
H	3.788922000	-0.423648000	-4.219669000
H	3.727539000	0.858859000	-2.984633000

REFERENCES

(S1) Macikenas, D.; Skrzypczak-Jankun, E.; Protasiewicz, J. A New Class of Iodonium Ylides Engineered as Soluble Primary Oxo and NitreneSources. *J. Am. Chem. Soc.* **1999**, *121* (30), 7164–7165.

(S2) Lim, M.; Rohde, J.; Stubna, A.; Bukowski, M.; Costas, M.; Ho, R.; Munck, E.; Nam, W.; Que, L. An FeIV=O complex of a tetradentate tripodal nonheme ligand. *Proc. Natl. Acad. Sci.* **2003**, *100* (7), 3665–3670.

(S3) Biswas, A. N.; Puri, M.; Meier, K.; Oloo, W.; Rohde, G.; Bominaar, E.; Münck, E.; Que, L. Modeling TauD-*J*: A High-Spin Nonheme Oxoiron(IV) Complex with High Reactivity toward C–H Bonds. *J. Am. Chem. Soc.* **2015**, *137* (7), 2428–2431.

(S4) Brandsma, L.; van Sodingen, J.; Andringa, H. A Procedure for the Preparation of Pure 1,4-Cyclohexadiene from Benzene. *Synth. Commun.* **1990**, *20*, 2165−2168.

(S5) Zhang, J.; Grills, D. C.; Huang, K.-W.; Fujita, E.; Bullock, R. M. Carbon-to-Metal Hydrogen Atom Transfer: Direct Observation Using Time-Resolved Infrared Spectroscopy. *J. Am. Chem. Soc.* **2005**, *127*, 15684–15685.

(S6) Becke, A. Density-functional thermochemistry. III. The role of exact exchange. *J. Chem. Phys.* **1993**, *98*, 5648–5652.

(S7) Lee, C. ; Yang, W.; Parr, R. Development of the Colle-Salvetti correlation-energy formula into a functional of the electron density. *Phys. Rev. B* **1988**, *37*, 785–789.
